# Global Priority Conservation Areas in the Face of 21^st^ Century Climate Change

**DOI:** 10.1371/journal.pone.0054839

**Published:** 2013-01-24

**Authors:** Junsheng Li, Xin Lin, Anping Chen, Townsend Peterson, Keping Ma, Monika Bertzky, Philippe Ciais, Valerie Kapos, Changhui Peng, Benjamin Poulter

**Affiliations:** 1 State Key Laboratory of Environmental Criteria and Risk Assessment, Chinese Research Academy of Environmental Sciences, Beijing, China; 2 College of Water Sciences, Beijing Normal University, Beijing, China; 3 Department of Ecology and Evolutionary Biology, Princeton University, Princeton, New Jersey, United States of America; 4 Biodiversity Institute, The University of Kansas, Lawrence, Kansas, United States of America; 5 Institute of Botany, The Chinese Academy of Sciences, Beijing, China; 6 United Nations Environment Programme World Conservation Monitoring Centre, Cambridge, United Kingdom; 7 Laboratoire des Sciences du Climat et de l’Environnement (LSCE), Unité Mixte (UM) de Commissariat à l’Énergie Atomique et aux Énergies Alternatives (CEA), Centre National de la Recherche Scientifique (CNRS) and Université de Versailles Saint Quentin Yvelines (UVSQ), Gif sur Yvette, France; 8 Department of Zoology, University of Cambridge, Cambridge, United Kingdom; 9 Department of Biology Sciences, Institute of Environment Sciences, University of Quebec, Montreal, Quebec, Canada; 10 Laboratory for Ecological Forecasting and Global Change, College of Forestry, Northwest Agriculture and Forestry University, Yangling, Shaanxi, China; Cirad, France

## Abstract

In an era when global biodiversity is increasingly impacted by rapidly changing climate, efforts to conserve global biodiversity may be compromised if we do not consider the uneven distribution of climate-induced threats. Here, via a novel application of an aggregate Regional Climate Change Index (RCCI) that combines changes in mean annual temperature and precipitation with changes in their interannual variability, we assess multi-dimensional climate changes across the “Global 200” ecoregions – a set of priority ecoregions designed to “achieve the goal of saving a broad diversity of the Earth’s ecosystems” – over the 21^st^ century. Using an ensemble of 62 climate scenarios, our analyses show that, between 1991–2010 and 2081–2100, 96% of the ecoregions considered will be likely (more than 66% probability) to face moderate-to-pronounced climate changes, when compared to the magnitudes of change during the past five decades. Ecoregions at high northern latitudes are projected to experience most pronounced climate change, followed by those in the Mediterranean Basin, Amazon Basin, East Africa, and South Asia. Relatively modest RCCI signals are expected over ecoregions in Northwest South America, West Africa, and Southeast Asia, yet with considerable uncertainties. Although not indicative of climate-change impacts *per se*, the RCCI-based assessment can help policy-makers gain a quantitative and comprehensive overview of the unevenly distributed climate risks across the G200 ecoregions. Whether due to significant climate change signals or large uncertainties, the ecoregions highlighted in the assessment deserve special attention in more detailed impact assessments to inform effective conservation strategies under future climate change.

## Introduction

The rapidly changing climate has significantly impacted global biodiversity during recent decades [1], and is likely to play an increasing role in biodiversity loss over the longer term [2]. Nevertheless, current global conservation prioritization schemes are mainly based on factors such as irreplaceability, vulnerability or threats from habitat loss [3]. Exposure of biodiversity to climate change, however, is rarely incorporated in conservation priority assessments, which may compromise conservation investments in priority conservation areas [4], [5]. As climate-induced threats to biodiversity are unevenly distributed in space and time, evaluation of future climate risks faced by the priority conservation areas could lead to reallocation of globally flexible funding and resources.

Several studies have attempted to develop indicators to assess the unevenly-distributed magnitudes of climate change at global or regional scales, based on changes in mean climate conditions, climate variability, frequencies of extreme events, or aggregations of these variables [4], [6]–[15]. Among those studies, a few recent ones focused on the magnitudes of climate change experienced by particular taxa or biologically distinct regions [4], [11]–[15], which could have important implications for climate adaptation and biodiversity conservation under future climate change. For example, Beaumont *et al*. (2011) [14] evaluated future climate change exposure of the “Global 200” ecoregions - a set of priority ecoregions designed to “achieve the goal of saving a broad diversity of the Earth’s ecosystems” [16] - during the 21^st^ century. Their findings suggested that tropical and subtropical ecoregions might be particularly vulnerable to future climate change, given that ecoregions at lower latitudes will be more likely to face “extreme” local temperatures compared to those at higher latitudes [14]. However, their evaluation was based on shifts in individual climatic factors (monthly mean temperature or precipitation), and did not incorporate changes in climate variability at any timescale. A more comprehensive assessment of climate risks should aggregate multi-dimensional future climate changes faced by these priority conservation areas.

In this paper, via a novel application of a Regional Climate Change Index (RCCI), an indicator integrating changes in mean temperature and precipitation with their interannual variability [6], we evaluate the climate-change exposure of 143 terrestrial and 53 freshwater ecoregions included in the “Global 200” (hereafter “G200”). Our assessment is based on differences in climate variables between the recent decade (1991−2010) and the end of the 21^st^ century (2081−2100), with outputs from an ensemble of 62 general circulation model (GCM) × green house gas (GHG) emission scenario combinations. To account for the inter-model agreements on the sign and magnitude of climate change, differently from the original approach used by Giorgi (2006) to calculate the RCCI [6], we estimate changes in component climatic factors from each of the 62 GCM × GHG emission scenario combinations separately rather than averaging over them. The changes in the multi-dimensional climate space reflected by the RCCI and its component climatic factors, together with degrees of model consensus on these changes, enable a better understanding of the unevenly-distributed climate risks across the G200 ecoregions, with significant implications for effective conservation strategies under the changing climate.

## Results and Discussion

Projected changes in mean temperature between 1991−2010 and 2081−2100 show a clear warming trend. The average temperature is projected to rise by 1.7 to 5.0°C across the 196 ecoregions, compared to the increase of 0 to 1.4°C over the past five decades, with strongest warming expected in ecoregions at high northern latitudes (Table S1, Figure S2A). Furthermore, future warming trends are projected to be stronger in dry seasons than in wet seasons (Figure S2B, C). Future precipitation changes, on the other hand, show high spatial heterogeneity and between-model variability (Table S1, Figure S2D−F). Generally, the likelihood of precipitation increase is high in ecoregions at high northern latitudes, East Africa, and South Asia, while precipitation decline is expected in ecoregions of the Mediterranean Basin, Central America, the Andes, South Africa, Madagascar, and Australia, particularly during dry seasons. The local decreases in precipitation, combined with warming-induced increased evapotranspiration, would further elevate moisture stress on terrestrial and freshwater ecosystems.

**Figure 1:**
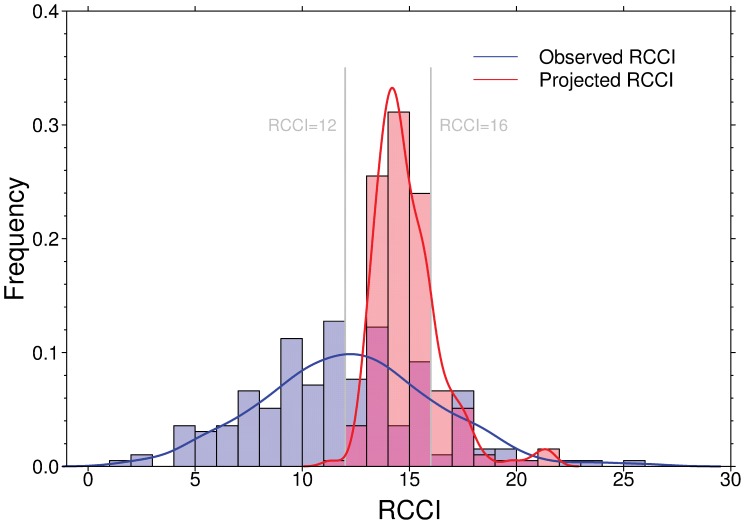
Frequency distributions of observed and projected Regional Climate Change Index (RCCI) across 196 G200 ecoregions. The observed RCCI (blue bars and solid line) is based on differences in climate conditions between 1961−1980 and 1991−2009, generated from Climate Research Unit (CRU) TS 3.1 datasets; the projected RCCI (red bars and solid line) is based on differences in climate conditions between 1991−2010 and 2081−2100, generated from the ensemble of 62 GCM × GHG emission scenario combinations. The grey vertical lines represent the 50^th^ and 80^th^ percentile of observed RCCI (i.e., RCCI = 12 and RCCI = 16), indicating moderate and pronounced climate change, respectively.

To combine changes in mean temperature and precipitation with changes in their interannual variability, we used the RCCI to summarize the relative climate-change exposure for each ecoregion. With an observed RCCI range between 2 and 26 over the past 5 decades (see *Materials and Methods*), we take the 50^th^ and 80^th^ percentile (i.e., RCCI = 12 and RCCI = 16) as indicative of “moderate” and “pronounced” climate change, respectively (Figure 1). The RCCI analyses from the ensemble of 62 GCM × GHG emission scenario combinations show that, throughout the 196 G200 ecoregions, 188 (96%) are predicted to have a mean RCCI ≥12, and a greater than 66% ensemble probability of at least “moderate” climate change (RCCI ≥12) by the end of the 21st century. A total of 14 (7%) ecoregions are predicted to have a mean RCCI ≥16, and at least a 66% ensemble probability of “pronounced” climate change (RCCI ≥16) (Table S2). The magnitude of climate change projected by the end of the 21^st^ century in general exceeds that experienced during the past five decades (Figure 1), implying that the climate-driven biodiversity consequences that have been observed recently [1], [17], [18] may be further amplified and accelerated.

**Figure 2:**
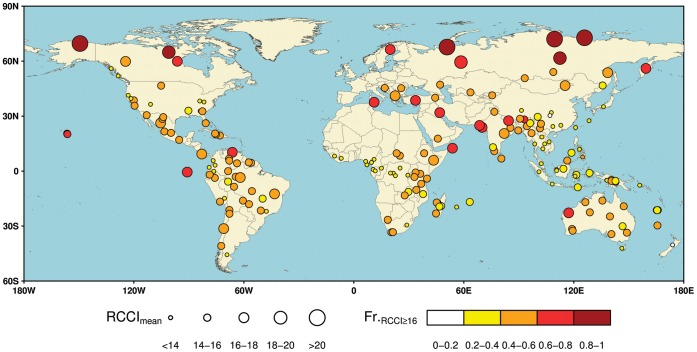
The spatial distributions of Regional Climate Change Index (RCCI) across 196 G200 ecoregions. Based on differences in climate conditions between 1991−2010 and 2081−2100 generated from the ensemble of 62 GCM × GHG emission scenario combinations, the relative climate-change exposure of each G200 ecoregion is indicated by the multi-model mean RCCI (RCCI_mean_, illustrated as the size of the symbol) and the proportion of GCM × GHG emission scenario combinations with RCCI ≥16 (Fr._RCCI≥16_, illustrated as the color of the symbol).

**Figure 3:**
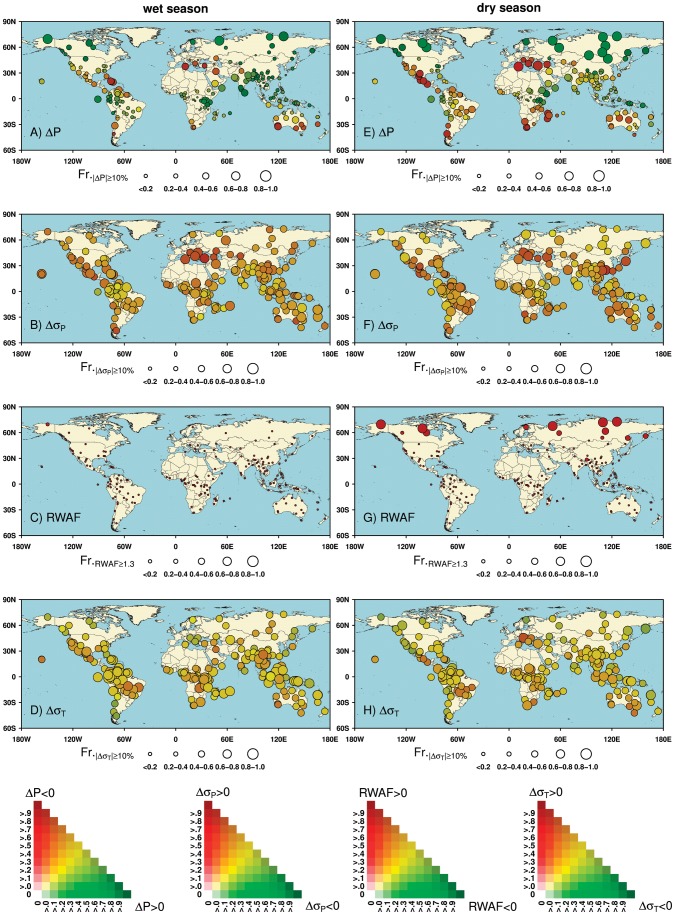
Changes in component climatic factors of Regional Climate Change Index (RCCI) across 196 G200 ecoregions. A) Wet season ΔP; B) wet season Δσ_P_; C) wet season RWAF; D) wet season Δσ_T_; E) dry season ΔP; F) dry season Δσ_P_; G) dry season RWAF; H) dry season Δσ_T_. The calculation is based on differences in climate conditions between 1991–2010 and 2081–2100, generated from the ensemble of 62 GCM × GHG emission scenario combinations. The changing magnitude of each component climatic factor is indicated by the proportion of GCM × GHG emission scenario combinations with the absolute value of the corresponding integer “n” ≥2 (illustrated as the size of the symbol). The changing direction is indicated by the proportion of combinations where an increase or decrease is projected to occur (illustrated as the color of the symbol).

The values of RCCI are unevenly distributed across the globe. The strongest regional climate change signals are expected in high northern latitudes across North America and Eurasia, especially tundra and taiga ecoregions in the Arctic and sub-Arctic. Six out of 10 ecoregions in this area have mean RCCI values exceeding 18, with 70% to 90% GCM × GHG emission scenario combinations predicting pronounced climate change (e.g., the *Chukhote Coastal Tundra*, *Taimyr and Russian Coastal Tundra*, *Alaskan North Slope Coastal Tundra*, *Canadian Low Arctic Tundra*, and *Central and Eastern Siberian Taiga* ecoregions; Figure 2, Table S2). The pronounced RCCI signals in the Arctic and sub-Arctic ecoregions are attributed to remarkable changes in both mean climate conditions and climate variability. Particularly, a strong warming trend is expected over the 21^st^ century, with average dry-season (November to April; Figure S5) warming of up to 6.9±2.7°C for the *Chukhote Coastal Tundra* ecoregion and wet-season (June to November; Figure S5) warming of up to 3.8±1.4°C for the *Alaskan North Slope Coastal Tundra* ecoregion (Figure 3, Table S1, Figure S2). Rapid high-latitude warming and concurrent increases in precipitation may favor lengthening of the growing season and northward expansion of temperate species and ecosystems [19], [20], but impacts on survival of key species adapted to this harsh and highly variable environment are uncertain [21], [22]. Many of these species, especially Arctic endemics, are particularly vulnerable to competitive stress from more southerly species, and have limited potential to escape through poleward shifts due to geometric constraints of the northernmost region on Earth (e.g. the displacement of Arctic foxes by invading red foxes in northern Norway [23]). Given generally low biodiversity and limited functional redundancy among species, loss of key species in Arctic and sub-Arctic regions could have cascading effects and precipitate irreversible changes in ecosystem dynamics, impacting ecosystem services and indigenous people depending on them [21], [22]. As our current knowledge of the climate-change impacts on the Arctic and sub-Arctic biodiversity remains limited, future work is required for better understanding of the complex processes involved under climate threats [22]. While a comparatively large proportion of the Arctic and sub-Arctic is already under protection [24], there is still an urgent need to develop appropriate strategies to mitigate climate-induced threats in these ecoregions given the potentially severe risks of rapid warming.

In mid-latitudes, the most dramatic climate changes are expected in the G200 ecoregions of the Mediterranean Basin. For the *Mediterranean Forests, Woodlands and Scrub*, *Anatolian Freshwater*, and *Balkan Rivers & Streams* ecoregions, the mean RCCI values are between 17 and 18, with a 60% to 80% probability of pronounced climate change (Figure 2, Table S2). The relatively strong climate change signals for these ecoregions are mainly contributed by a substantial precipitation decline and a large increase in precipitation variability, especially during dry seasons (Figure 3E–F, Table S1, Figure S2D–F). For example, in the *Mediterranean Forests, Woodlands and Scrub* ecoregion, the ensemble of 62 GCM × scenario combinations consistently indicates a decrease in dry season precipitation, by as much as −23.3±11.9% (Table S1). As one of the world’s five regions within the mediterranean biome, the Mediterranean Basin has unique climatic and edaphic conditions, and supports high levels of species richness and endemism [25]. However, ecoregions here are susceptible to large biodiversity loss because of their exceptional sensitivity to environmental changes [26]. The warmer and drier climates in the coming decades will increase drought stress and fire risks in these ecoregions, leading to changes in community structure and shifts in the distribution of typical tree species [27], [28]. Moreover, the impact of climate change on biodiversity may be further exacerbated by non-climatic stressors such as land conversion and biological invasions [29], [30]. Despite these ecoregions’ biological importance and the imminent threats they face, the land area currently under formal protection is less than 1%, compared to a global average of 12% [31], reinforcing the need to expand the network of protected areas to facilitate the migration and adaptation of Mediterranean species under future climate change [31], [32]. Besides, remarkable precipitation declines are also expected in other Mediterranean-type ecoregions such as the *Fynbos* and *Chilean Matorral* ecoregion (Figure 3E–F, Table S1, Figure S2D–F), which are also renowned for their outstanding endemism richness but under pressure from anthropogenic impacts [30].

In the tropical and subtropical regions, comparatively strong climate changes are expected over G200 ecoregions of the Amazon Basin, East Africa, and South Asia (Figure 2). In the Amazon Basin, a region which sustains almost 60% of the world’s remaining tropical rainforest and *ca.* 25% of all terrestrial species [33], the *Coastal Venezuela Montane Forests*, *Amazon River & Flooded Forests*, and *Atlantic Dry Forests* ecoregions have mean RCCI values between 16 and 18, with pronounced climate change predicted by no less than 55% of the GCM × scenario combinations (Figure 2, Table S2). The relatively strong RCCI signals for ecoregions in the Amazon Basin are attributed primarily to the notable changes in seasonal precipitation as well as temperature and precipitation variability (Figure 3). However, regarding the magnitudes and directions of these changes, there are large variations across different scenarios and climate models (Figure 3, Table S1). The projected changes in dry season precipitation over the *Amazon River & Flooded Forests* ecoregion, for example, range widely from −56% to +36%. Despite the considerable uncertainty in the climate projections, a trend toward less dry-season precipitation and intensified drought risk is projected to occur over most ecoregions located in the Amazon Basin (Figure 3, Table S1). More pronounced dry seasons could increase the risk of forest dieback and savannization [34], [35], and may lead to losses of forest species, shifts in community composition, and erosion of ecosystem services, especially when interacting with fire disturbance and human activities such as deforestation, commercial logging, and expansion of infrastructure [36], [37]. In these ecoregions, taking conservation actions to minimize non-climatic pressures and increase their climate resilience may be of particular importance, mainly through a sustainable way to manage socio-economic development as well as effective financial incentives [38]. Other subtropical and tropical ecoregions that will face significant climate change include the *Galapagos Islands Scrub* ecoregion off the northwest coast of South America, the *Socotra Island Desert*, and *Horn of Africa Acacia Savannas* ecoregions in East Africa, and the *Rann of Kutch Flooded Grasslands*, and *Indus River Delta* ecoregions in South Asia (Figure 2, Table S2).

Based on multi-model mean RCCI values as well as degrees of inter-model consistency, our assessment highlights pronounced future climate change over ecoregions at high northern latitudes, followed by ecoregions of the Mediterranean Basin, Amazon Basin, East Africa, and South Asia. The strength of the RCCI-based assessment lies in, on the one hand, the integration of changes in mean annual temperature and precipitation, as well as their interannual variability, which presents a more comprehensive picture of future climate risks compared to that on a basis of only one or two climate factors (e.g., [11], [14], [15]). Moreover, break-down analyses of individual RCCI component climatic factors allow for further identification of the main contributors of climate change signals, as well as their directions and magnitudes. The vulnerability inferred from the RCCI and key aspects of climate change may have significant implications for climate-change impact prediction and conservation, as illustrated earlier for the highlighted ecoregions with strong RCCI signals. Even for ecoregions that face relatively moderate climate change implied by the RCCI indicator, severe climate impacts on those components of biodiversity that are vulnerable to strong and consistent changes in individual climatic factors are still likely to occur. For instance, two ecoregions of Madagascar, the *Madagascar Forests and Shrublands*, and *Madagascar Freshwater Ecosystem* ecoregions, are projected to experience a decrease in dry season precipitation by more than 10%, with the likelihood of precipitation decline as high as 87% (Table S1). Although their RCCI signals are relatively small (Figure 2, Table S2), these ecoregions with unparalleled levels of endemism are very likely to face intense drought risks in the coming decades, imperiling the unique species and their habitats that are already threatened by decades of deforestation and forest fragmentation [39], [40].

On the other hand, the multiple GHG emission scenarios and climate models used in RCCI estimation enable quantification of inter-model agreements on future climate change for each ecoregion, i.e. the degree to which we are confident in a result from the RCCI-based assessment. This emphasizes the uncertainties of scenarios and climate models that may induce substantial between-model variations in the climate change assessment, which was, however, not adequately addressed in earlier similar studies (e.g., [6], [13]). We notice considerable variations across the ensemble of 62 GCM × GHG emission scenario combinations in terms of the RCCI and changes in component climatic factors, especially for ecoregions with relatively low RCCI signals (Figure S3). This great variability arises from uncertainties in both GHG emission scenarios and climate models (Figure S4). Ecoregions where future climate change is project to be relatively mild but highly uncertain may still have chance to face severe climate risks under the “worst-case” scenarios, such as those located in Northwest South America (e.g., the *South American Pacific Mangroves*, and *Upper Amazon Rivers & Streams* ecoregions), West Africa (e.g., the *Guinean Moist Forests*, and *Upper Guinea Rivers & Streams* ecoregions), and Southeast Asia (e.g., the *Annamite Range Moist Forests*, *Sumatran Islands Lowland and Montane Forests*, *Peninsular Malaysia Lowland and Montane Forests* ecoregions; Figure 2, Figure S3). The considerable between-model disagreement in the RCCI-based assessment not only underlies the importance of model improvements in climate projections, but also suggests flexible approaches that mix a portfolio of different adaptation and mitigation strategies are required for different situations as climates and environments continue to shift [41], [42].

Compared to former studies based on the original or adapted RCCI indicators, for example, Giorgi (2006) [6] and Bonebrake & Mastrandrea (2010) [13], our assessment for the G200 ecoregions generally produces a similar spatial pattern of future climate change exposure. All three studies suggest that northern high latitudes and the Mediterranean region are expected to face pronounced climate change by the end of the 21^st^ century. However, significant differences also exist between our assessment and the two earlier ones. For example, in both Giorgi (2006) [6] and Bonebrake & Mastrandrea (2010) [13], the Amazon Basin, South Asia, and North Australia are expected to face mild or moderate climate change, while our study indicates that at least some ecoregions within the three regions will be exposed to strong climate change (e.g., the *Coastal Venezuela Montane Forests*, *Amazon River & Flooded Forests*, and *Atlantic Dry Forests* ecoregions of the Amazon Basin; the *Rann of Kutch Flooded Grasslands*, and *Indus River Delta* ecoregions of South Asia; the *Carnavon Xeric Shrubs* ecoregion of North Australia). The discrepancies between our study and the two former ones may arise from differences in focal areas (196 G200 ecoregions vs. 26 distinct regions; see Figure S1 of this paper and Figure 1 of Giorgi (2006) [6]), choices of climate models (see Table S4 of this paper and Table 1 of Giorgi & Bi (2005) [43]) and baseline periods (1991–2010 v.s. 1961–1980) that could preclude the inter-comparisons. Moreover, we estimated changes in climatic factors and thus the RCCI from climate projections of each GCM × scenario combination separately, rather than averaging over models and scenarios as done by Giorgi (2006) [6] and Bonebrake & Mastrandrea (2010) [13] (see *Materials and Methods*), which may also contribute to the discrepancy between our results and those of the two earlier studies. As stated above, our approach accounts for uncertainties from scenarios and climate models, and enables quantification of inter-model agreements on the results of the RCCI-based assessment.

Several other studies have also attempted to develop indicators to assess magnitudes of future climate change across distinct regions based on different principles and climatic factors (e.g. [4], [7], [10]–[12], [14], [15]). A recent study by Beaumont et al. (2011) also evaluated future climate risks faced by the G200 ecoregions, but using changes in projected climatic conditions relative to baseline variability [14]. In contrast with our result, they suggested that tropical and subtropical ecoregions are likely to be exposed to more severe climate risks rather than boreal/taiga and tundra ecoregions at high northern latitudes, based on the fact that the relative temperature change compared to the historical temperature variation in tropical and subtropical regions is larger than that of high latitude regions [14]. However, it should be noted that the magnitude of warming in the tropical and subtropical regions is substantially smaller than that in the high latitudes. Further, changes in other climatic factors (e.g., precipitation, climate variability) may pose effects no less than that of mean temperature change and should be considered to present a broader picture of future climate risks. Across the studies that developed a number of indicators to assess the future climate change, the ‘hotspots’ emerging with high climate risks may differ from each other with varying degrees, revealing differences in the indicator structures (including component climatic factors, weighting factors, level of aggregation, etc.) and principles to formulate them, apart from inconsistency of analyzed periods, selection of climate models, and focal areas [7]. While these studies provide complementary information on the complex picture of unevenly distributed climate-induced threats, discrepancies among them suggest that the choice of indicators in climate-change assessment need to be cautious according to specific conservation and management targets.

A few caveats should be noted in our analyses. First, the thresholds we used to define “moderate” and “pronounced” climate change (RCCI = 12 and RCCI = 16, respectively), as well as the assignment of integer factor “n” in RCCI calculation (Eq. 1), are somehow subjectively determined based on the distributions of changes rather than critical points beyond which significant transitions will occur [6]. However, since our focus is the relative magnitudes of climate change across the G200 ecoregions, changes in these thresholds and thus RCCI values for specific ecoregions would not alter the overall pattern and results of intercomparison among them. Second, the RCCI only evaluates changes in mean temperature and precipitation with their variability, on an interannual basis. As climate change will exert impacts on biodiversity at different time scales [44], additional indicators that reveal climate variations and extremes from diurnal to multi-decadal time scales should also be considered to give a more comprehensive picture of future climate risks. Third, we weighted each of the 62 GCM × GHG emission scenario combinations equally, which may obscure much of the uncertainty behind GCMs and scenarios [45]. Differential weights should be given to the ensemble members in further analyses, depending on the future socioeconomic pathways and GCM performance at global and regional scales [46], [47].

It also needs to be emphasized that the RCCI and related metrics do not directly translate into climate-change impacts on biodiversity. These metrics can be regarded as robust indicators of future climate-change exposure, yet detailed assessments of climate-change impacts on biodiversity need to consider other aspects of vulnerability: climate sensitivity and adaptive capacity [2], [48]. For example, tropical species living in warm, aseasonal climates may be vulnerable to even small temperature changes due to limited thermal tolerance and acclimation capacities evolved under relatively uniform temperature regimes [49], [50]. As a consequence, direct biodiversity impacts of climate warming may be more severe in the tropics despite their slower warming rate compared to higher-latitude areas [51]. Understanding climate-change impacts on biodiversity is further complicated by the diversity of ecological and evolutionary responses at different organisational levels [1], interactions of climate-change effects with other stressors (e.g., land conversion, overexploitation, invasive species, fire, pathogens) [52], and feedbacks and cascading impacts [48]. This complexity calls for integrated assessments combining different approaches, including long-term observations, manipulative experiments, and modeling [2], which is beyond the scope of this study. However, although not indicative of climate-change impacts *per se*, the RCCI-based assessment can help policy-makers gain a quantitative overview of future climate change across the G200 ecoregions. The ecoregions highlighted in our study, whether due to significant climate risks or high levels of uncertainty, deserve further in-depth impact assessments to inform effective conservation strategies under climate change.

### Conclusions

Our RCCI-based assessment of climate-change exposure of the G200 ecoregions suggests that, by the end of the 21^st^ century, 96% of the G200 ecoregions are likely (more than 66% probability) to face climate change that is considered moderate-to-pronounced compared to changes experienced over the past five decades. Northern high-latitude ecoregions will see the most pronounced climate change, followed by ecoregions of the Mediterranean Basin, Amazon Basin, East Africa, and South Asia. Relatively modest climate change is expected over ecoregions in Northwest South America, West Africa, and Southeast Asia, yet with considerable uncertainty. The integration of multi-dimensional climate changes in the RCCI-based assessment enables a quantitative and comprehensive overview of future climate risks across the G200 ecoregions, while the estimation of inter-model agreements on the RCCI and related climatic metrics gives the degrees to which we are confident in the results. The highlighted ecoregions, together with their vulnerability inferred from the RCCI and key aspects of climate change, deserve special attention in more detailed impact assessments to inform effective conservation strategies. Particularly, for ecoregions with considerable inter-model disagreement, a portfolio of flexible approaches that integrates different adaptation and mitigation strategies is essential to cope with the uncertain future.

The RCCI used in our study accounts for shifts in mean temperature and precipitation with their variability on an interannual basis. Climate variabilities and extremes on other time scales, which may pose additional threats to biodiversity, should also be incorporated. While indicators of different structures provide complementary information on the complex pattern of unevenly distributed climate-induced threats, caution should be taken to choose appropriate indicators in the light of specific conservation and management targets. Note that assessment based on any of the indicators does not directly translate into the vulnerability to climate change. Detailed assessments of climate-change impacts on biodiversity should also consider climate sensitivity and adaptive capacity, as well as interactions between climatic and non-climatic stressors. This calls for integrated assessments that combine different data sources and approaches, including long-term observations, manipulative experiments, and modeling.

As delays in negotiating a new global agreement for the post-Kyoto period appear to push the world towards more pessimistic IPCC scenarios, climate change impacts on biodiversity and people may become even more severe. Future priority-setting and allocation of limited conservation resources should consider the unevenly-distributed climate-induced threats to inform effective conservation policies and actions in coming decades. Furthering our understanding of future climate change exposure across space and time is an important first step in the identification and design of appropriate conservation strategies that can help adapt to and/or mitigate the impacts of such changes on biodiversity, and ultimately people.

## Materials and Methods

### Climate Datasets

To estimate future climate-change exposure, we used climate projections for the 21^st^ century from an ensemble of 23 general circulation models (GCMs) from the CMIP3 multi-model datasets (https://esg.llnl.gov:8443/index.jsp) simulated under three IPCC SRES scenarios (B1, A1B, A2) [53], producing 62 GCM × GHG emission scenario combinations in total (note that for some GCMs, datasets were available under only 1 or 2 emission scenarios; Table S4). Simulated climate data for the period of 1991−2000 were extracted from GCMs for the 20^th^ century scenario (20c3m simulation). For each GCM × GHG emission scenario combination, different realizations were averaged before further analyses (Table S4). We also calculated the observed climate-change exposure based on differences in observed climate conditions between the baseline period (1961−1980) and recent decades (1991−2009), and compared the projected climate-change exposure with it. Observed climate data were obtained from Climate Research Unit (CRU) TS 3.1 datasets at the resolution of 0.5° × 0.5° [54]. All the data were first gridded to a common 0.1° grid and then averaged over each G200 ecoregion.

### Definition of the RCCI

The RCCI (Regional Climate Change Index) is an aggregate index that integrates changes in four climate factors: mean annual precipitation (ΔP, % of the baseline value), mean annual surface air temperature (RWAF, Regional Warming Amplification Factor, i.e., change in mean annual temperature relative to the mean annual global warming), and their interannual variability (Δσ_P_ and Δσ_T_, each in % of the baseline value) [6]. The RCCI is defined and calculated as follows (Eq.1; ref. [6]):
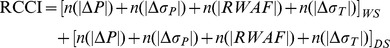
(1)where any change in each climatic factor is assigned an integer value “n” between 0 and 4 according to the absolute value of change (Table S3), resulting in an RCCI range between 0 and 32. The division between wet season (WS) and dry season (DS) for each ecoregion is based on Giorgi & Bi (2005) (Figure S5; ref. [43]). Higher RCCI values represent higher climate-change exposure for any ecoregion. Note that the values of the aggregate RCCI index are always positive, yet the direction of change in each component climatic factor could be positive or negative.

### Observed RCCI

Based on differences in observed climate conditions between 1961−1980 and 1991−2009, we calculated the observed RCCI index for each of the 196 G200 ecoregions over the past five decades. The observed RCCI ranges from 2 to 26, with a median of 12 (Figure 1). We took the 50^th^ and 80^th^ percentile of observed RCCI (i.e., RCCI = 12 and RCCI = 16) as indicative of “moderate” and “pronounced” climate change, respectively. The magnitude of future climate change was evaluated in reference to the criteria.

### Projected RCCI

Based on differences in projected climate conditions between 1991−2010 and 2081−2100, we calculated the RCCI to estimate future climate-change exposure for each of the 196 G200 ecoregions. We explored three IPCC SRES climate scenarios (B1, A1B, A2) [53], and produced the RCCI from the ensemble of 62 GCM × GHG emission scenario combinations. To account for the inter-model agreements on the sign and magnitude of climate change, rather than calculating changes in climatic factors and thus RCCI by averaging over models and scenarios as done by Giorgi (2006) [6], we produced the RCCI from climate projections of each GCM × scenario combination separately. Relative climate-change exposure of each ecoregion was summarized as the multi-model mean RCCI and probability of GCM × GHG emission scenario combinations predicting RCCI ≥16. To analyze contributions of component climatic factors and potential impacts on ecoregions, we also examined the magnitude and direction of change in each one individually (Figure 3). We weighted each GCM × scenario combination equally.

All analyses were performed in R.

## Supporting Information

Figure S1 **The spatial distribution of 196 “Global 200” ecoregions, grouped by biomes.** **A**) Terrestrial “Global 200” ecoregions; **B**) freshwater “Global 200” ecoregions. The figure in the bracket indicates the number of ecoregions within each biome. The two maps are adapted from Figure 1 and Figure 2 of Ref. [16], respectively.
(TIF)

Figure S2 **Changes in mean temperature and precipitation across 196 G200 ecoregions between 1991−2010 and 2081−2100.** The calculation is based on multi-model averages from the ensemble of 62 GCM × GHG emission scenario combinations. **A**) ΔT for all year; **B**) wet season ΔT; **C**) dry season ΔT; **D**) ΔP for all year; **E**) wet season ΔP; **F**) dry season ΔP.
(TIF)

Figure S3 **Coefficients of variations (CV) of RCCI across 62 GCM × GHG emission scenario combinations for 196 G200 ecoregions.** RCCI is calculated based on differences in climate conditions between 1991−2010 and 2081−2100.
(TIF)

Figure S4 **The spatial distributions of RCCI across 196 G200 ecoregions under three different GHG emission scenarios.** The calculation is based on differences in climate conditions between 1991−2010 and 2081−2100. Generated from the ensemble of 20, 23, and 19 GCMs for **A**) SRES B1; **B**) SRES A1B; and **C**) SRES A2, respectively, the relative climate-change exposure of each G200 ecoregion is indicated by the multi-model mean RCCI (RCCI_mean_, illustrate as the size of the symbol) and the proportion of GCMs with RCCI ≥16 (Fr._RCCI≥16_, illustrated as the color of the symbol).
(TIF)

Figure S5 **Definitions of wet seasons for different regions.** Dry seasons are the remaining six months of a year. For each G200 ecoregion, wet and dry seasons are identified according to its geographic location. This map is drawn based on Ref. [43].
(TIF)

Table S1 **Changes in mean climate conditions across 196 G200 ecoregions.** The calculation is based on differences in climate conditions between 1991−2010 and 2081−2100, generated from an ensemble of 62 GCM × GHG emission scenario combinations. Changes in temperature and precipitation are calculated on an annual basis (ΔT, ΔP) as well as for wet seasons (ΔT_wet_, ΔP_wet_) and dry seasons (ΔT_dry_, ΔP_dry_). For precipitation, the proportions of GCM × scenario combinations with ΔP>0 (abbreviated as Fr. (ΔP>0), Fr.( ΔP_wet_>0), Fr (ΔP_dry_>0)) are also given to indicate likelihoods of precipitation increase. Standard deviations (s.d.) are calculated to account for variations across different GCM × GHG emission scenario combinations.
(DOC)

Table S2 **Observed and projected RCCI for 196 G200 ecoregions.** The observed RCCI (RCCI_obs_) is based on differences in climate conditions between 1961−1980 and 1991−2009, generated from Climate Research Unit (CRU) TS 3.1 datasets; the projected RCCI is based on differences in climate conditions between 1991−2010 and 2081−2100, generated from an ensemble of 62 GCM × GHG emission scenario combinations. The relative magnitude of projected RCCI is measured by the multi-model mean RCCI (RCCI_mean_) and the proportions of GCM × GHG emission scenario combinations with RCCI≥12 (the 50^th^ percentile of RCCI_obs_) and RCCI≥16 (the 80^th^ percentile of RCCI_obs_), respectively (abbreviated as Fr.(RCCI≥12) and Fr.(RCCI≥16)).
(DOC)

Table S3 **Value of the factor n in the definition of the RCCI.** Any change in each climatic factor is assigned an integer value “n” between 0 and 4 according to the absolute value of change. Note that small changes below a certain threshold do not contribute to the index (n = 0) and that larger changes are weighted more heavily (i.e., the factor n doubles from each category to the next). This table is adapted from Table 1 of Ref. [6].
(DOC)

Table S4 **The ensemble of 23 General Circulation Models (GCMs) used in this study.** Figures in brackets indicate the number of different realizations for a GCM × GHG emission scenario combination.
(DOC)

## References

[1] Parmesan C (2006) Ecological and evolutionary responses to recent climate change. Annu Rev Ecol Evol Syst 37: 637–669.

[2] Dawson TP, Jackson ST, House JI, Prentice IC, Mace GM (2011) Beyond predictions: biodiversity conservation in a changing climate. Science 332: 53–58. 21454781 10.1126/science.1200303

[3] Brooks TM, Mittermeier RA, da Fonseca GAB, Gerlach J, Hoffmann M, et al (2006) Global biodiversity conservation priorities. Science 313: 58–61. 16825561 10.1126/science.1127609

[4] Iwamura T, Wilson KA, Venter O, Possingham HP (2010) A climatic stability approach to prioritizing global conservation investments. PLoS ONE 5: e15103. 21152095 10.1371/journal.pone.0015103 PMC2994894

[5] Araújo MB, Alagador D, Cabeza M, Nogués-Bravo D, Thuiller W (2011) Climate change threatens European conservation areas. Ecol Lett 14: 484–492. 21447141 10.1111/j.1461-0248.2011.01610.x PMC3116148

[6] Giorgi F (2006) Climate change hot-spots. Geophys Res Lett 33: L08707.

[7] Baettig MB, Wild M, Imboden DM (2007) A climate change index: where climate change may be most prominent in the 21^st^ century. Geophys Res Lett 34: L01705.

[8] Diffenbaugh NS, Giorgi F, Raymond L, Bi X (2007) Indicators of 21^st^ century socioclimatic exposure. Proc Natl Acad Sci USA 104: 20195–20198. 18077324 10.1073/pnas.0706680105 PMC2154407

[9] Williams JW, Jackson ST, Kutzbach JE (2007) Projected distributions of novel and disappearing climates by 2100 AD. Proc Natl Acad Sci USA 104: 5738–5742. 17389402 10.1073/pnas.0606292104 PMC1851561

[10] Diffenbaugh NS, Giorgi F, Pal JS (2008) Climate change hotspots in the United States. Geophys Res Lett 35: L16709.

[11] Loarie SR, Duffy PB, Hamilton H, Asner GP, Field CB, et al (2009) The velocity of climate change. Nature 462: 1052–1055. 20033047 10.1038/nature08649

[12] Ackerly DD, Loarie SR, Cornwell WK, Weiss SB, Hamilton H, et al (2010) The geography of climate change: implications for conservation biogeography. Divers Distrib 16: 476–487.

[13] Bonebrake TC, Mastrandrea MD (2010) Tolerance adaptation and precipitation changes complicate latitudinal patterns of climate change impacts. Proc Natl Acad Sci USA 107: 12581–12586. 20616038 10.1073/pnas.0911841107 PMC2906544

[14] Beaumont LJ, Pitman A, Perkins S, Zimmermann NE, Yoccoz NG, et al (2011) Impacts of climate change on the world’s most exceptional ecoregions. Proc Natl Acad Sci USA 108: 2306–2311. 21262825 10.1073/pnas.1007217108 PMC3038729

[15] Sandel B, Arge L, Dalsgaard B, Davies RG, Gaston KJ, et al (2011) The influence of late Quaternary climate-change velocity on species endemism. Science 334: 660–664. 21979937 10.1126/science.1210173

[16] Olson DM, Dinerstein E (1998) The Global 200: a representation approach to conserving the Earth’s most biologically valuable ecoregions. Cons Biol 12: 502–515.

[17] Walther G-R, Post E, Convey P, Menzel A, Parmesan C, et al (2002) Ecological responses to recent climate change. Nature 416: 389–395. 11919621 10.1038/416389a

[18] Walther G-R (2010) Community and ecosystem responses to recent climate change. Phil Trans R Soc B 365: 2019–2024. 20513710 10.1098/rstb.2010.0021 PMC2880129

[19] Tape KEN, Sturm M, Racine C (2006) The evidence for shrub expansion in Northern Alaska and the Pan-Arctic. Glob Change Biol 12: 686–702.

[20] Høye TT, Post E, Meltofte H, Schmidt NM, Forchhammer MC (2007) Rapid advancement of spring in the High Arctic. Curr Biol 17: R449–R451. 17580070 10.1016/j.cub.2007.04.047

[21] Post E, Forchhammer MC, Bret-Harte MS, Callaghan TV, Christensen TR, et al (2009) Ecological dynamics across the Arctic associated with recent climate change. Science 325: 1355–1358. 19745143 10.1126/science.1173113

[22] Gilg O, Kovacs KM, Aars J, Fort J, Gauthier G, et al (2012) Climate change and the ecology and evolution of Arctic vertebrates. Ann NY Acad Sci 1249: 166–190. 22329928 10.1111/j.1749-6632.2011.06412.x

[23] Killengreen ST, Ims RA, Yoccoz NG, Bråthen KA, Henden J-A, et al (2007) Structural characteristics of a low Arctic tundra ecosystem and the retreat of the Arctic fox. Biol Cons 135: 459–472.

[24] Usher MB, Callaghan TV, Gilchrist G, Heal B, Juday GP, et al.. (2005) Principles of conserving the Arctic’s biodiversity. In: Symon C, Arris L, Heal B, editors. Arctic Climate Impact Assessment. Cambridge: Cambridge University Press. 540–591.

[25] Keeley JE, Swift CC (1995) Biodiversity and ecosystem functioning in Mediterranean-climate California. In: Davis GW, Richardson DM, editors. Mediterranean Type Ecosystems: The Function of Biodiversity. Heidelberg: Springer-Verlag. 122–183.

[26] Sala OE, Chapin FS, Armesto JJ, Berlow E, Bloomfield J, et al (2000) Global biodiversity scenarios for the year 2100. Science 287: 1770–1774. 10710299 10.1126/science.287.5459.1770

[27] Mouillot F, Rambal S, Joffre R (2002) Simulating climate change impacts on fire frequency and vegetation dynamics in a Mediterranean-type ecosystem. Glob Change Biol 8: 423–437.

[28] Schröter D, Cramer W, Leemans R, Prentice IC, Araújo MB, et al (2005) Ecosystem service supply and vulnerability to global change in Europe. Science 310: 1333–1337. 16254151 10.1126/science.1115233

[29] Gritti ES, Smith B, Sykes MT (2006) Vulnerability of Mediterranean Basin ecosystems to climate change and invasion by exotic plant species. J Biogeogr 33: 145–157.

[30] Underwood EC, Viers JH, Klausmeyer KR, Cox RL, Shaw MR (2009) Threats and biodiversity in the mediterranean biome. Divers Distrib 15: 188–197.

[31] Klausmeyer KR, Shaw MR (2009) Climate change, habitat loss, protected areas and the climate adaptation potential of species in mediterranean ecosystems worldwide. PLoS ONE 4: e6392. 19641600 10.1371/journal.pone.0006392 PMC2712077

[32] Underwood EC, Klausmeyer KR, Cox RL, Busby SM, Morrison SA, et al (2009) Expanding the global network of protected areas to save the imperiled mediterranean biome. Cons Biol 23: 43–52. 10.1111/j.1523-1739.2008.01072.x 18950475

[33] Dirzo R, Raven PH (2003) Global state of biodiversity and loss. Annu Rev Environ Resour 28: 137–167.

[34] Zelazowski P, Malhi Y, Huntingford C, Sitch S, Fisher JB (2011) Changes in the potential distribution of humid tropical forests on a warmer planet. Phil Trans R Soc A 369: 137–160. 21115517 10.1098/rsta.2010.0238

[35] Cox PM, Betts RA, Collins M, Harris PP, Huntingford C, et al (2004) Amazonian forest dieback under climate-carbon cycle projections for the 21^st^ century. Theor Appl Climat 78: 137–156.

[36] Laurance WF, Nascimento HEM, Laurance SG, Andrade A, Ribeiro JELS, et al (2006) Rapid decay of tree-community composition in Amazonian forest fragments. Proc Natl Acad Sci USA 103: 19010–19014. 17148598 10.1073/pnas.0609048103 PMC1682011

[37] Davidson EA, de Araujo AC, Artaxo P, Balch JK, Brown IF, et al (2012) The Amazon basin in transition. Nature 481: 321–328. 22258611 10.1038/nature10717

[38] Malhi Y, Roberts JT, Betts RA, Killeen TJ, Li W, et al (2008) Climate change, deforestation, and the fate of the Amazon. Science 319: 169–172. 18048654 10.1126/science.1146961

[39] Harper GJ, Steininger MK, Tucker CJ, Juhn D, Hawkins F (2007) Fifty years of deforestation and forest fragmentation in Madagascar. Environ Cons 34: 325–333.

[40] Hannah L, Dave R, Lowry PP, Andelman S, Andrianarisata M, et al (2008) Climate change adaptation for conservation in Madagascar. Biol Lett 4: 590–594. 18664414 10.1098/rsbl.2008.0270 PMC2610084

[41] Millar CI, Stephenson NL, Stephens SL (2007) Climate change and forests of the future: managing in the face of uncertainty. Ecol Appl 17: 2145–2151. 18213958 10.1890/06-1715.1

[42] Canadell JG, Raupach MR (2008) Managing forests for climate change mitigation. Science 320: 1456–1457. 18556550 10.1126/science.1155458

[43] Giorgi F, Bi X (2005) Updated regional precipitation and temperature changes for the 21st century from ensembles of recent AOGCM simulations. Geophys Res Lett 32: L21715.

[44] Jones RN (2001) An environmental risk assessment/management framework for climate change impact assessments. Nat Hazards 23: 197–230.

[45] Tebaldi C, Smith RL, Nychka D, Mearns LO (2005) Quantifying uncertainty in projections of regional climate change: A bayesian approach to the analysis of multimodel ensembles. J Clim 18: 1524–1540.

[46] Tebaldi C, Knutti R (2007) The use of the multi-model ensemble in probabilistic climate projections. Phil Trans R Soc A 365: 2053–2075. 17569654 10.1098/rsta.2007.2076

[47] Fordham DA, Wigley TML, Brook BW (2011) Multi-model climate projections for biodiversity risk assessments. Ecol Appl 21: 3317–3331.

[48] Williams SE, Shoo LP, Isaac JL, Hoffmann AA, Langham G (2008) Towards an integrated framework for assessing the vulnerability of species to climate change. PLoS Biol 6: e325. 10.1371/journal.pbio.0060325 PMC2605927 19108608

[49] Janzen DH (1967) Why mountain passes are higher in the Tropics. Am Nat 101: 233–249.

[50] Ghalambor CK, Huey RB, Martin PR, Tewksbury JJ, Wang G (2006) Are mountain passes higher in the tropics? Janzen’s hypothesis revisited. Int Comp Biol 46: 5–17. 10.1093/icb/icj003 21672718

[51] Tewksbury JJ, Huey RB, Deutsch CA (2008) Putting the heat on tropical animals. Science 320: 1296–1297. 18535231 10.1126/science.1159328

[52] Brook BW, Sodhi NS, Bradshaw CJA (2008) Synergies among extinction drivers under global change. Trends Ecol Evol 23: 453–460. 18582986 10.1016/j.tree.2008.03.011

[53] IPCC (2007) Climate Change 2007: The Physical Science Basis. Contribution of Working Group I to the Fourth Assessment Report of the Intergovernmental Panel on Climate Change; Solomon S, Qin D, Manning M, Chen Z, Marquis M et al., editors. Cambridge: Cambridge University Press. 996 p.

[54] Mitchell TD, Jones PD (2005) An improved method of constructing a database of monthly climate observations and associated high-resolution grids. Int J Climatol 25: 693–712.

