# Supplementary figures and images for: Global Priority Conservation Areas in the Face of 21st Century Climate Change
Source: PLoS One. 2013 Jan 24;8(1):e54839. doi: 10.1371/journal.pone.0054839 (PMC3554607; doi:10.1371/journal.pone.0054839)

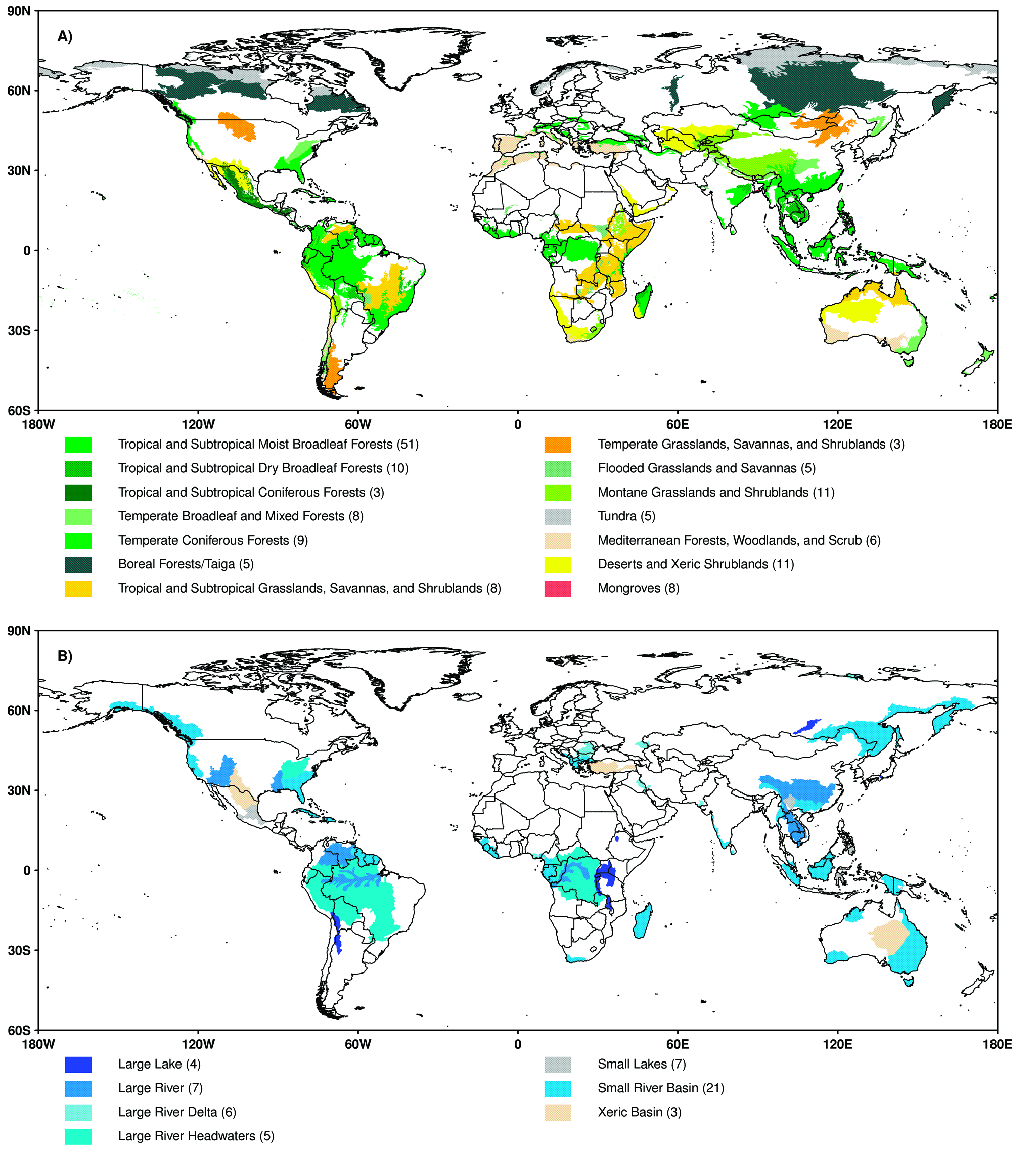

Supplement: Figure S1 — The spatial distribution of 196 “Global 200” ecoregions, grouped by biomes. A) Terrestrial “Global 200” ecoregions; B) freshwater “Global 200” ecoregions. The figure in the bracket indicates the number of ecoregions within each biome. The two maps are adapted from Figure 1 and Figure 2 of Ref. [16], respectively. (TIF) [file pone.0054839.s001.tif]

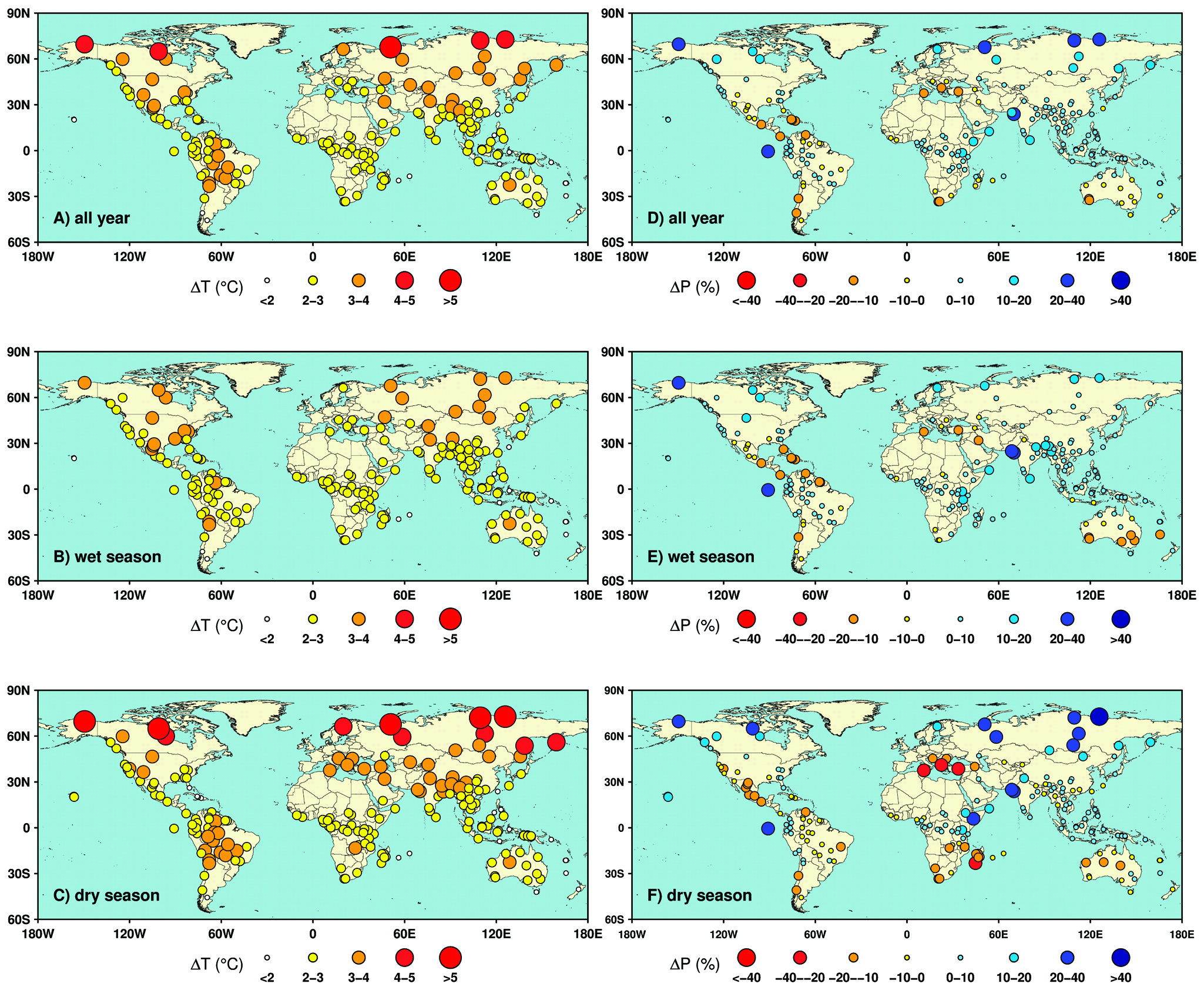

Supplement: Figure S2 — Changes in mean temperature and precipitation across 196 G200 ecoregions between 1991−2010 and 2081−2100. The calculation is based on multi-model averages from the ensemble of 62 GCM × GHG emission scenario combinations. A) ΔT for all year; B) wet season ΔT; C) dry season ΔT; D) ΔP for all year; E) wet season ΔP; F) dry season ΔP. (TIF) [file pone.0054839.s002.tif]

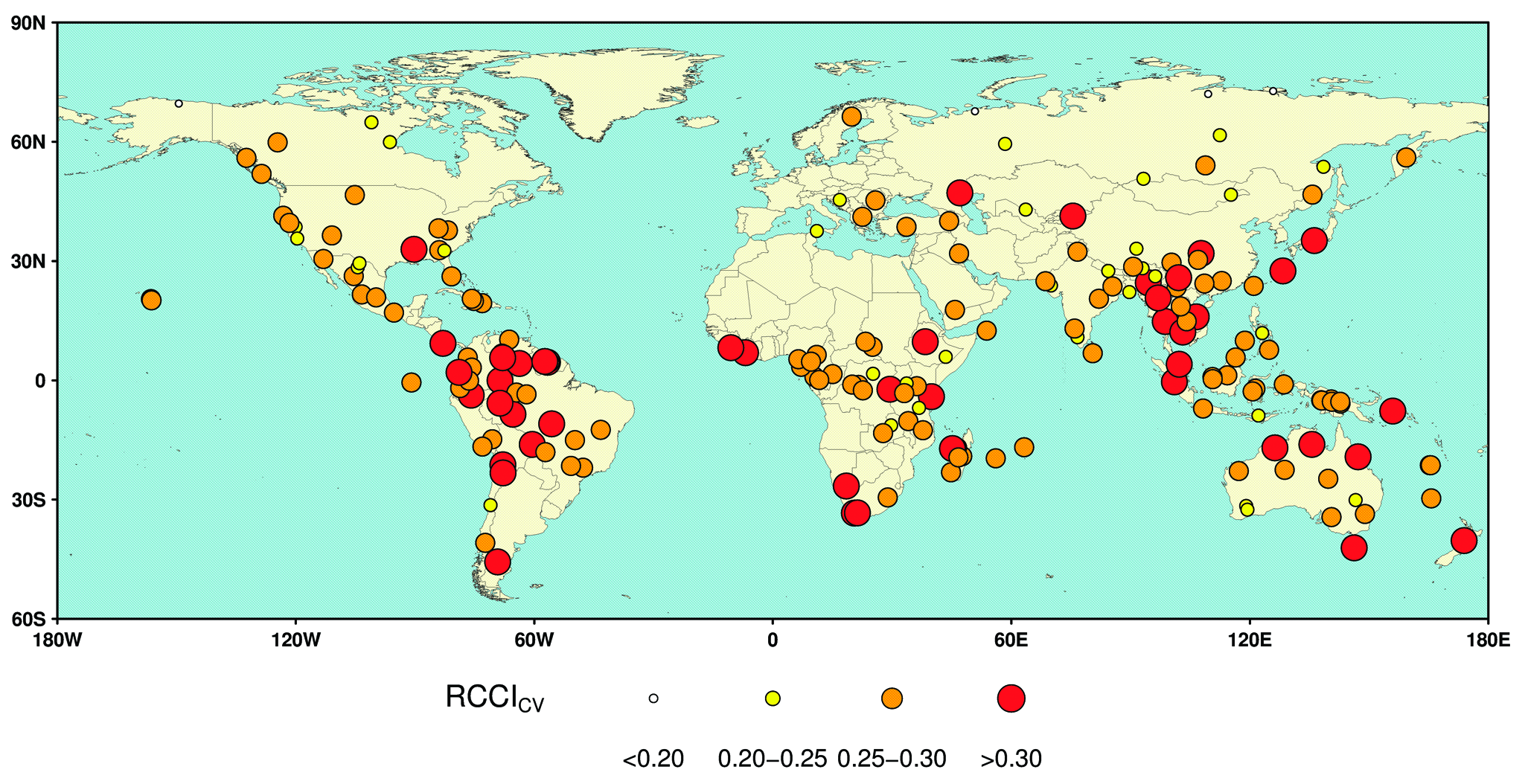

Supplement: Figure S3 — Coefficients of variations (CV) of RCCI across 62 GCM × GHG emission scenario combinations for 196 G200 ecoregions. RCCI is calculated based on differences in climate conditions between 1991−2010 and 2081−2100. (TIF) [file pone.0054839.s003.tif]

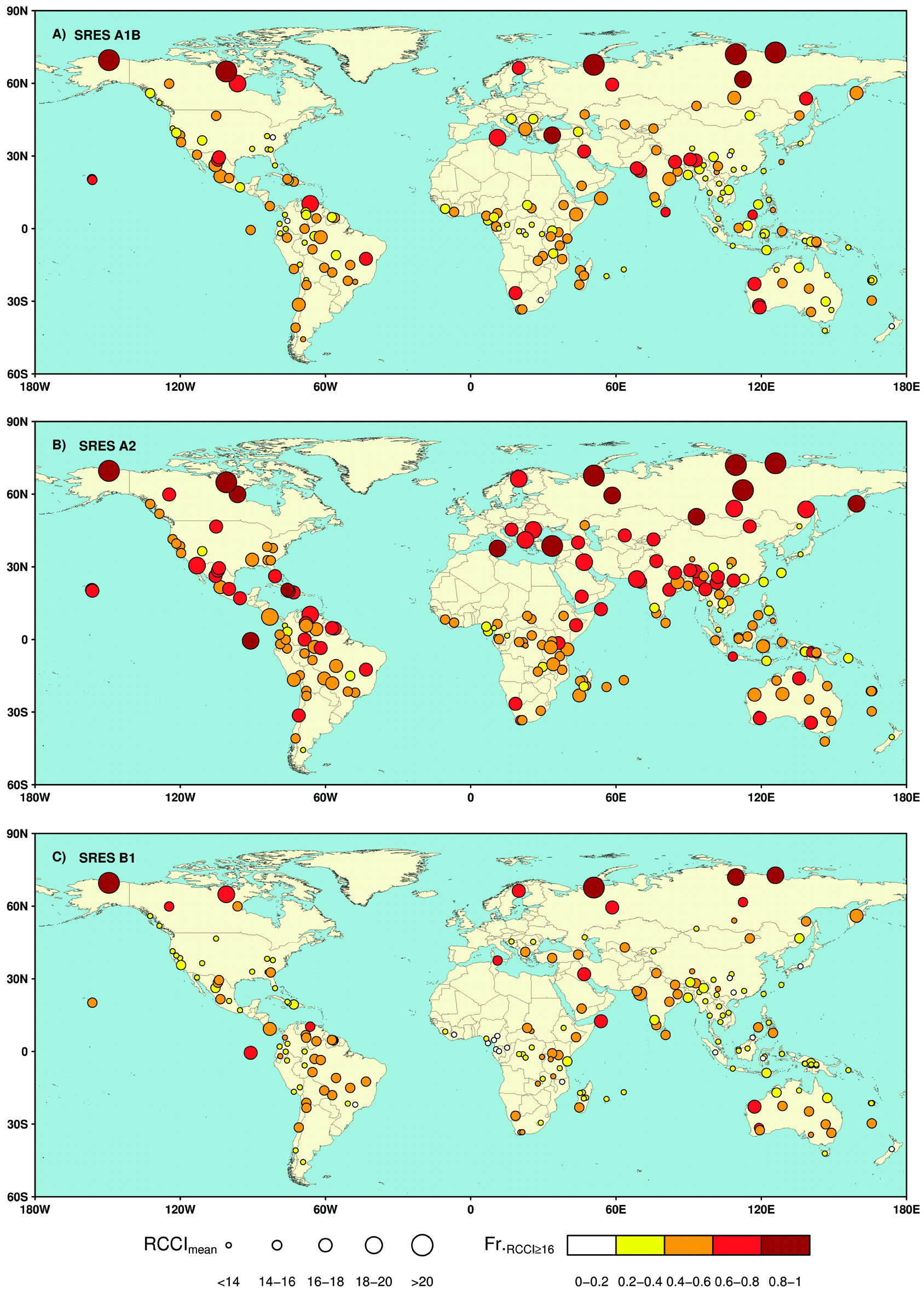

Supplement: Figure S4 — The spatial distributions of RCCI across 196 G200 ecoregions under three different GHG emission scenarios. The calculation is based on differences in climate conditions between 1991−2010 and 2081−2100. Generated from the ensemble of 20, 23, and 19 GCMs for A) SRES B1; B) SRES A1B; and C) SRES A2, respectively, the relative climate-change exposure of each G200 ecoregion is indicated by the multi-model mean RCCI (RCCImean, illustrate as the size of the symbol) and the proportion of GCMs with RCCI ≥16 (Fr.RCCI≥16, illustrated as the color of the symbol). (TIF) [file pone.0054839.s004.tif]

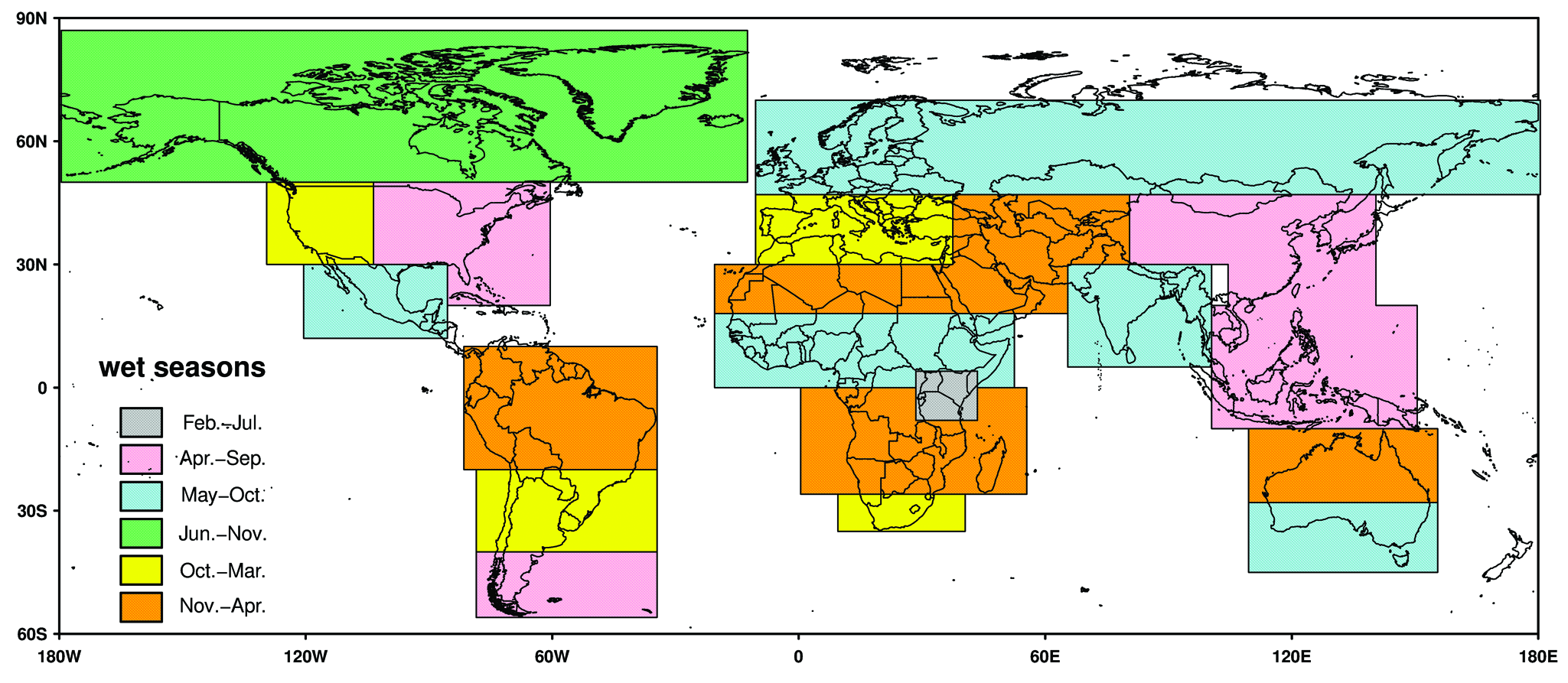

Supplement: Figure S5 — Definitions of wet seasons for different regions. Dry seasons are the remaining six months of a year. For each G200 ecoregion, wet and dry seasons are identified according to its geographic location. This map is drawn based on Ref. [43]. (TIF) [file pone.0054839.s005.tif]
