# Supplementary material for: Global Priority Conservation Areas in the Face of 21st Century Climate Change
Source: PLoS One. 2013 Jan 24;8(1):e54839. doi: 10.1371/journal.pone.0054839 (PMC3554607; doi:10.1371/journal.pone.0054839)
Supplement: Table S1 — Changes in mean climate conditions across 196 G200 ecoregions. The calculation is based on differences in climate conditions between 1991−2010 and 2081−2100, generated from an ensemble of 62 GCM × GHG emission scenario combinations. Changes in temperature and precipitation are calculated on an annual basis (ΔT, ΔP) as well as for wet seasons (ΔTwet, ΔPwet) and dry seasons (ΔTdry, ΔPdry). For precipitation, the proportions of GCM × scenario combinations with ΔP>0 (abbreviated as Fr. (ΔP>0), Fr.( ΔPwet>0), Fr (ΔPdry>0)) are also given to indicate likelihoods of precipitation increase. Standard deviations (s.d.) are calculated to account for variations across different GCM × GHG emission scenario combinations. (DOC) [file pone.0054839.s006.doc]

**Table S1 Changes in mean climate conditions across 196 G200 ecoregions.** The calculation is based on differences in climate conditions between 1991−2010 and 2081−2100, generated from an ensemble of 62 GCM × GHG emission scenario combinations. Changes in temperature and precipitation are calculated on an annual basis (ΔT, ΔP) as well as for wet seasons (ΔT_wet_, ΔP_wet_) and dry seasons (ΔT_dry_, ΔP_dry_). For precipitation, the proportions of GCM × scenario combinations with ΔP>0 (abbreviated as Fr. (ΔP>0), Fr.(ΔP_wet_>0), Fr (ΔP_dry_>0), respectively) are also given to indicate likelihoods of precipitation increase. Standard deviations (s.d.) are calculated to account for variations across different GCM × GHG emission scenario combinations.

| G200_ID | G200 Ecoregion | ΔT (℃) | ΔT_wet_ (℃) | ΔT_dry_ (℃) | ΔP (%) | Fr. (ΔP>0) (%) | ΔP_wet_ (%) | Fr.(ΔP_wet_>0)  (%) | ΔP_dry_ (%) | Fr. (ΔP_dry_>0) (%) |
| --- | --- | --- | --- | --- | --- | --- | --- | --- | --- | --- |
| 1 | Guinean Moist Forests | 2.4±0.8 | 2.3±0.8 | 2.5±0.9 | -0.4±7.0 | 45.2 | -0.2±7.2 | 46.8 | -2.7±11.5 | 30.6 |
| 2 | Congolian Coastal Forests | 2.4±0.8 | 2.4±0.7 | 2.4±0.8 | 2.4±6.5 | 56.5 | 2.7±7.4 | 59.7 | 2.1±7.9 | 59.7 |
| 3 | Cameroon Highlands Forests | 2.5±0.8 | 2.4±0.8 | 2.6±0.9 | 3.4±7.7 | 61.3 | 4.0±8.5 | 59.7 | -0.1±9.7 | 50.0 |
| 4 | Northeastern Congo Basin Moist  Forests | 2.6±0.9 | 2.6±0.9 | 2.6±0.9 | 4.3±5.6 | 79.0 | 4.3±6.8 | 74.2 | 4.2±7.5 | 71.0 |
| 5 | Central Congo Basin Moist Forests | 2.6±0.9 | 2.6±0.9 | 2.7±0.9 | 3.2±5.9 | 66.1 | 3.3±7.7 | 66.1 | 3.0±6.7 | 66.1 |
| 6 | Western Congo Basin Moist Forests | 2.5±0.8 | 2.5±0.8 | 2.5±0.9 | 2.5± 5.2 | 59.7 | 2.4±5.8 | 67.7 | 2.2±7.1 | 61.3 |
| 7 | Albertine Rift Montane Forests | 2.6±0.9 | 2.7±0.9 | 2.6±0.8 | 6.7±7.7 | 83.9 | 7.4±8.0 | 83.9 | 6.3±8.7 | 79.0 |
| 8 | East African Coastal Forests | 2.3±0.7 | 2.3±0.8 | 2.3±0.7 | 7.1±12.0 | 80.6 | 7.5±12.2 | 82.3 | 6.9±15.3 | 71.0 |
| 9 | Eastern Arc Montane Forests | 2.5±0.8 | 2.4±0.8 | 2.5±0.8 | 7.9±9.3 | 88.7 | 11.3±10.9 | 95.2 | 4.8±11.5 | 66.1 |
| 10 | Madagascar Forests and Shrublands | 2.3±0.7 | 2.3±0.8 | 2.3±0.7 | -0.9±7.2 | 43.5 | 1.7±8.4 | 54.8 | -14.0±13.5 | 12.9 |
| 11 | Seychelles and Mascarenes Moist  Forests | 1.8±0.6 | 1.9±0.6 | 1.8±0.6 | -0.7±7.6 | 41.9 | 1.8±10.0 | 54.8 | -6.7±8.5 | 12.9 |
| 12 | Sulawesi Moist Forests | 2.0±0.7 | 2.1±0.7 | 2.0±0.7 | 4.0±7.5 | 74.2 | 3.6±11.0 | 66.1 | 4.2±6.1 | 75.8 |
| 13 | Moluccas Moist Forests | 1.9±0.6 | 1.9±0.6 | 1.9±0.6 | 6.3±7.4 | 82.3 | 7.1±9.6 | 74.2 | 5.4±8.2 | 72.6 |
| 14 | Southern New Guinea Lowland  Forests | 2.1±0.7 | 2.1±0.7 | 2.1±0.7 | 7.2±6.4 | 90.3 | 6.9±8.9 | 79.0 | 7.4±6.3 | 90.3 |
| 15 | New Guinea Montane Forests | 2.1±0.7 | 2.1±0.7 | 2.0±0.7 | 8.0±7.2 | 90.3 | 7.1±8.9 | 77.4 | 8.3±7.4 | 87.1 |
| 16 | Solomons-Vanuatu-Bismarck Moist  Forests | 1.9±0.6 | 1.9±0.6 | 1.9±0.6 | 8.3±6.8 | 87.1 | 7.3±8.2 | 80.6 | 9.0±7.1 | 88.7 |
| 17 | Queensland Tropical Forests | 2.1±0.7 | 2.1±0.7 | 2.1±0.7 | -1.7±10.8 | 51.6 | -0.2±12.0 | 56.5 | -7.4±15.4 | 38.7 |
| 18 | New Caledonia Moist Forests | 1.9±0.6 | 1.9±0.6 | 1.8±0.6 | 0.7±10.7 | 45.2 | 2.9±12.4 | 53.2 | -4.5±12.7 | 32.3 |
| 19 | Lord Howe and Norfolk Island  Forests | 1.9±0.6 | 1.7±0.6 | 2.0±0.7 | -5.2±11.2 | 32.3 | -13.1±12.1 | 14.5 | 3.7±13.7 | 61.3 |
| 20 | Southwestern Ghats Moist Forest | 2.3±0.7 | 2.2±0.7 | 2.3±0.8 | 7.0±8.4 | 80.6 | 8.4±9.0 | 82.3 | 2.4±17.9 | 59.7 |
| 21 | Sri Lankan Moist Forest | 2.0±0.7 | 2.0±0.7 | 2.0±0.7 | 10.3±9.0 | 91.9 | 14.7±12.6 | 93.5 | 3.9±10.9 | 64.5 |
| 22 | North Indochina Subtropical Moist  Forests | 2.5±0.8 | 2.4±0.8 | 2.6±0.8 | 6.2±7.9 | 77.4 | 7.6±7.5 | 85.5 | 0.0±15.3 | 50.0 |
| 23 | Southeast China-Hainan Moist  Forests | 2.5±0.8 | 2.4±0.8 | 2.5±0.8 | 4.8±5.4 | 83.9 | 7.1±5.1 | 91.9 | -1.4±12.3 | 48.4 |
| 24 | Taiwan Montane Forests | 2.0±0.6 | 2.0±0.6 | 2.0±0.6 | 2.0±8.1 | 59.7 | 6.2±8.3 | 79.0 | -5.1±11.7 | 33.9 |
| 25 | Annamite Range Moist Forests | 2.2±0.7 | 2.3±0.8 | 2.2±0.7 | 2.7±6.0 | 77.4 | 4.0±6.9 | 75.8 | 0.3±12.6 | 54.8 |
| 26 | Sumatran Islands Lowland and  Montane Forests | 2.2±0.7 | 2.2±0.7 | 2.1±0.7 | 3.5±5.4 | 72.6 | 4.3±6.5 | 79.0 | 2.8±6.0 | 69.4 |
| 27 | Philippines Moist Forests | 2.0±0.6 | 2.1±0.7 | 2.0±0.6 | 3.0±5.4 | 74.2 | 3.8±5.0 | 79.0 | 2.0±9.8 | 67.7 |
| 28 | Palawan Moist Forests | 1.9±0.6 | 2.0±0.6 | 1.9±0.6 | 3.1±6.2 | 72.6 | 4.8±6.4 | 83.9 | 1.1±10.5 | 61.3 |
| 29 | Kayah-Karen/Tenasserim Moist  Forests | 2.4±0.7 | 2.2±0.7 | 2.5±0.8 | 4.3±4.8 | 82.3 | 6.3±5.7 | 93.5 | -6.1±16.8 | 40.3 |
| 30 | Peninsular Malaysia Lowland and  Montane Forests | 2.2±0.7 | 2.2±0.7 | 2.1±0.7 | 3.8±5.2 | 74.2 | 6.5±4.8 | 90.3 | 1.2±8.6 | 54.8 |
| 31 | Borneo Lowland and Montane Forests | 2.2±0.7 | 2.2±0.7 | 2.1±0.7 | 6.2±6.8 | 79.0 | 6.9±6.7 | 85.5 | 5.6±8.1 | 75.8 |
| 32 | Nansei Shoto Archipelago Forests | 2.0±0.6 | 2.0±0.6 | 2.0±0.7 | -0.3±5.8 | 53.2 | 4.4±6.1 | 77.4 | -6.7±8.3 | 27.4 |
| 33 | Eastern Deccan Plateau Moist Forests | 2.7±0.9 | 2.5±1.0 | 3.0±0.9 | 3.9±12.5 | 67.7 | 5.0±12.9 | 74.2 | 0.8±24.0 | 45.2 |
| 34 | Naga-Manapuri-Chin Hills Moist  Forests | 2.6±0.8 | 2.4±0.8 | 2.9±0.9 | 9.0±8.1 | 90.3 | 10.1±7.1 | 95.2 | 2.9±18.2 | 58.1 |
| 35 | Cardamom Mountains Moist Forests | 2.3±0.8 | 2.3±0.8 | 2.3±0.8 | 3.0±5.5 | 74.2 | 3.6±6.4 | 74.2 | 2.9±15.4 | 59.7 |
| 36 | Western Java Montane Forests | 2.1±0.7 | 2.1±0.7 | 2.1±0.7 | 1.1±8.2 | 53.2 | -1.9±13.0 | 46.8 | 3.0±7.5 | 64.5 |
| 37 | Greater Antillean Moist Forests | 2.0±0.6 | 2.0±0.6 | 2.0±0.6 | -12.0±14.2 | 12.9 | -19.4±17.7 | 12.9 | -3.5±12.6 | 45.2 |
| 38 | Talamancan-Isthmian Pacific Forests | 2.3±0.8 | 2.3±0.8 | 2.3±0.8 | -12.3±20.4 | 33.9 | -12.3±21.7 | 37.1 | -9.7±21.2 | 24.2 |
| 39 | Chocó-Darién Moist Forests | 2.4±0.8 | 2.4±0.8 | 2.4±0.8 | 0.9±6.5 | 53.2 | 2.6±9.9 | 59.7 | 0.1±6.9 | 46.8 |
| 40 | Northern Andean Montane Forests | 2.5±0.9 | 2.5±0.8 | 2.5±0.9 | 3.6±7.1 | 62.9 | 5.3±9.3 | 80.6 | 2.4±7.9 | 53.2 |
| 41 | Coastal Venezuela Montane Forests | 2.5±0.9 | 2.4±0.9 | 2.6±1.1 | -18.9±22.4 | 14.5 | -15.5±22.0 | 25.8 | -19.5±23.6 | 21.0 |
| 42 | Guianan Moist Forests | 2.6±0.9 | 2.5±0.9 | 2.6±0.9 | -8.9±17.9 | 27.4 | -12.9±20.0 | 25.8 | -7.0±18.7 | 29.0 |
| 43 | Napo Moist Forests | 2.8±1.0 | 2.6±0.9 | 3.0±1.2 | 5.5±7.6 | 82.3 | 6.3±7.6 | 85.5 | 4.2±11.6 | 66.1 |
| 44 | Rio Negro-Juruá Moist Forests | 2.9±1.2 | 2.8±1.1 | 3.0±1.3 | 3.0±10.1 | 66.1 | 5.5±12.8 | 72.6 | -0.3±12.2 | 53.2 |
| 45 | Guianan Highlands Moist Forests | 3.0±1.2 | 3.0±1.1 | 3.0±1.2 | -4.3±12.3 | 40.3 | 1.5±23.2 | 48.4 | -5.8±12.8 | 35.5 |
| 46 | Central Andean Yungas | 2.9±1.0 | 2.7±0.9 | 3.1±1.0 | 4.9±6.5 | 80.6 | 6.2±5.1 | 91.9 | 0.3±16.5 | 48.4 |
| 47 | Southwestern Amazonian Moist  Forests | 3.1±1.2 | 2.8±1.1 | 3.4±1.3 | 1.9±8.3 | 56.5 | 3.3±7.6 | 72.6 | -4.0±17.3 | 50.0 |
| 48 | Atlantic Forests | 2.5±0.8 | 2.4±0.9 | 2.5±0.8 | 2.1±7.6 | 71.0 | 3.1±7.4 | 69.4 | -0.3±15.8 | 48.4 |
| 49 | Southern Pacific Islands Forests | 1.8±0.6 | 1.8±0.6 | 1.8±0.6 | 2.7±9.6 | 69.4 | 4.4±10.9 | 80.6 | -0.3±11.0 | 48.4 |
| 50 | Hawaii Moist Forest | 2.0±0.7 | 1.9±0.7 | 2.0±0.7 | 5.0±20.4 | 51.6 | 0.3±12.1 | 51.6 | 11.7±46.9 | 40.3 |
| 51 | Madagascar Dry Forests | 2.3±0.7 | 2.3±0.8 | 2.3±0.8 | 1.7±8.0 | 58.1 | 3.4±8.9 | 64.5 | -15.2±17.0 | 14.5 |
| 52 | Nusu Tenggara Dry Forests | 2.0±0.6 | 2.0±0.6 | 2.0±0.6 | 0.4±9.0 | 56.5 | -2.5±15.4 | 41.9 | 2.0±6.8 | 66.1 |
| 53 | New Caledonia Dry Forests | 1.9±0.6 | 1.9±0.6 | 1.8±0.6 | 0.6±10.7 | 43.5 | 2.8±12.6 | 53.2 | -4.7±12.7 | 33.9 |
| 54 | Indochina Dry Forests | 2.3±0.8 | 2.4±0.8 | 2.3±0.8 | 3.9±4.9 | 83.9 | 4.6±5.6 | 83.9 | 1.6±14.0 | 53.2 |
| 55 | Chhota-Nagpur Dry Forests | 2.7±0.9 | 2.5±1.0 | 3.0±1.0 | 5.9±11.8 | 74.2 | 7.1±12.3 | 75.8 | 3.0±22.2 | 51.6 |
| 56 | Southern Mexican Dry Forests | 2.7±1.0 | 2.8±1.0 | 2.6±1.0 | -9.4±12.2 | 16.1 | -7.0±13.3 | 37.1 | -17.6±13.0 | 4.8 |
| 57 | Tumbesian-Andean Valleys Dry  Forests | 2.3±0.8 | 2.3±0.7 | 2.4±0.8 | 7.8±7.6 | 91.9 | 9.1±9.3 | 91.9 | 5.8±8.8 | 72.6 |
| 58 | Chiquitano Dry Forests | 3.1±1.1 | 2.8±1.1 | 3.3±1.1 | 0.4±9.3 | 59.7 | 1.8±9.5 | 61.3 | -7.5±23.8 | 30.6 |
| 59 | Atlantic Dry Forests | 2.7±0.9 | 2.7±1.0 | 2.6±0.9 | -2.9±13.1 | 48.4 | -1.5±13.7 | 50.0 | -18.1±23.6 | 21.0 |
| 60 | Hawaii Dry Forests | 2.0±0.7 | 2.0±0.7 | 2.0±0.8 | 5.7±21.7 | 50.0 | 0.7±12.5 | 51.6 | 13.0±51.2 | 40.3 |
| 61 | Sierra Madre Oriental and Occidental  Pine-Oak Forests | 3.0±1.0 | 3.1±1.1 | 2.8±1.1 | -9.1±11.5 | 19.4 | -4.4±13.0 | 33.9 | -17.6±11.9 | 1.6 |
| 62 | Greater Antillean Pine Forests | 2.0±0.7 | 2.1±0.7 | 2.0±0.7 | -10.3±12.4 | 14.5 | -17.1±16.1 | 9.7 | -2.3±9.8 | 41.9 |
| 63 | Mesoamerican Pine-Oak Forests | 2.7±1.0 | 2.8±1.0 | 2.5±0.9 | -11.1±13.9 | 12.9 | -10.5±15.3 | 22.6 | -14.9±12.9 | 4.8 |
| 64 | Eastern Asutralia Temperate Forests | 2.3±0.8 | 2.2±0.7 | 2.4±0.8 | -4.9±8.1 | 33.9 | -11.6±10.2 | 12.9 | 0.4±9.4 | 53.2 |
| 65 | Tasmanian Temperate Rainforests | 1.7±0.6 | 1.6±0.6 | 1.8±0.7 | -2.4±7.9 | 35.5 | 1.5±8.1 | 51.6 | -8.6±11.4 | 14.5 |
| 66 | New Zealand Temperate Forests | 1.9±0.6 | 1.8±0.6 | 1.9±0.7 | 0.7±3.5 | 62.9 | 1.4±3.8 | 62.9 | 0.0±6.3 | 59.7 |
| 67 | Eastern Himalayan Broadleaf and  Conifer Forests | 3.0±0.9 | 2.7±0.8 | 3.2±1.0 | 9.8±13.0 | 87.1 | 12.1±14.1 | 91.9 | 3.4±14.9 | 64.5 |
| 68 | Western Himalayan Temperate  Forests | 3.4±1.1 | 3.5±1.2 | 3.2±1.1 | 1.9±12.8 | 56.5 | -7.9±11.5 | 21.0 | 10.2±24.1 | 58.1 |
| 69 | Appalachian and Mixed Mesophytic  Forests | 2.9±1.1 | 3.2±1.3 | 2.7±1.0 | 5.0±7.3 | 77.4 | 3.3±9.8 | 69.4 | 7.1±8.7 | 80.6 |
| 70 | Southwest China Temperate Forests | 2.8±0.9 | 2.8±0.9 | 2.8±1.0 | 6.7±6.5 | 83.9 | 6.1±7.5 | 72.6 | 8.0±9.7 | 79.0 |
| 71 | Russian Far East Broadleaf and  Mixed Forests | 3.2±1.2 | 2.9±1.2 | 3.5±1.4 | 8.3±4.8 | 95.2 | 8.3±4.7 | 98.4 | 8.4±7.0 | 87.1 |
| 72 | Pacific Temperate Rainforests | 2.5±0.9 | 2.6±1.0 | 2.5±1.0 | 7.7±4.8 | 95.2 | 6.6±5.4 | 87.1 | 9.0±6.2 | 93.5 |
| 73 | Klamath-Siskiyou Coniferous Forests | 2.5±0.9 | 2.2±0.8 | 2.8±1.1 | 3.0±11.3 | 58.1 | 5.0±11.8 | 61.3 | -8.3±13.2 | 29.0 |
| 74 | Sierra Nevada Coniferous Forests | 2.9±1.0 | 2.5±0.9 | 3.2±1.2 | 1.9±14.0 | 48.4 | 4.3±14.8 | 54.8 | -10.8±15.5 | 25.8 |
| 75 | Southeastern Conifer and Broadleaf  Forests | 2.6±1.0 | 2.9±1.1 | 2.4±0.9 | 1.6±8.4 | 64.5 | 0.2±11.5 | 58.1 | 2.8±9.8 | 61.3 |
| 76 | Valdivian Temperate Rain Forests  / Juan Fernández | 1.9±0.6 | 1.6±0.5 | 2.2±0.7 | -11.4±6.8 | 3.2 | -8.6±5.9 | 6.5 | -16.5±10.5 | 4.8 |
| 77 | European-Mediterranean Montane  Forests | 2.9±1.0 | 2.5±0.9 | 3.2±1.2 | -5.6±6.8 | 21.0 | 1.2±5.6 | 62.9 | -12.7±10.9 | 8.1 |
| 78 | Caucasus-Anatolian-Hyrcanian Temperate Forests | 2.9±0.9 | 2.4±0.8 | 3.4±1.1 | -8.0±6.4 | 8.1 | -2.4±6.1 | 37.1 | -17.6±11.6 | 3.2 |
| 79 | Altai-Sayan Montane Forests | 3.3±1.3 | 3.3±1.2 | 3.4±1.5 | 8.9±5.1 | 98.4 | 3.4±6.9 | 71.0 | 18.6±7.5 | 100.0 |
| 80 | Hengduan Shan Conifer Forests | 2.9±0.9 | 2.7±0.9 | 3.1±1.0 | 7.2±6.8 | 85.5 | 6.8±6.8 | 87.1 | 9.0±9.7 | 83.9 |
| 81 | Muskwa/Slave Lake Boreal Forests | 3.1±1.1 | 2.8±1.1 | 3.5±1.3 | 11.2±6.5 | 98.4 | 9.9±7.3 | 93.5 | 13.2±7.4 | 96.8 |
| 82 | Canadian Boreal Taiga | 3.8±1.3 | 3.1±1.3 | 4.5±1.6 | 12.8±5.2 | 100.0 | 10.1±4.8 | 100.0 | 17.8±8.4 | 100.0 |
| 83 | Ural Mountains Taiga and Tundra | 3.8±1.5 | 3.0±1.3 | 4.5±1.8 | 12.7±5.6 | 100.0 | 6.4±6.2 | 82.3 | 20.9±9.9 | 100.0 |
| 84 | Central and Eastern Siberian Taiga | 3.9±1.5 | 3.1±1.3 | 4.7±1.9 | 14.3±5.0 | 100.0 | 9.9±4.8 | 95.2 | 26.6±12.4 | 100.0 |
| 85 | Kamchatka Taiga and Grasslands | 3.6±1.5 | 2.9±1.3 | 4.2±1.9 | 11.8±6.4 | 98.4 | 9.5±6.3 | 96.8 | 14.7±9.1 | 98.4 |
| 86 | Horn of Africa Acacia Savannas | 2.6±0.8 | 2.5±0.8 | 2.6±0.8 | 12.7±14.5 | 90.3 | 6.5±14.7 | 74.2 | 23.2±19.9 | 91.9 |
| 87 | East African Acacia Savannas | 2.5±0.8 | 2.5±0.9 | 2.5±0.8 | 12.2±11.8 | 93.5 | 13.0±13.2 | 87.1 | 11.8±14.0 | 90.3 |
| 88 | Central and Eastern Miombo  Woodlands | 2.8±0.9 | 2.6±0.9 | 2.9±1.0 | 3.0±5.4 | 71.0 | 4.1±5.5 | 77.4 | -5.3±11.3 | 33.9 |
| 89 | Sudanian Savannas | 2.8±0.9 | 2.8±1.0 | 2.8±0.9 | 2.6±7.2 | 61.3 | 1.9±8.0 | 56.5 | 4.5±10.5 | 62.9 |
| 90 | Northern Australia and Trans-Fly  Savannas | 2.5±0.8 | 2.5±0.8 | 2.6±0.8 | 1.2±8.0 | 50.0 | 1.9±8.4 | 56.5 | -7.3±17.0 | 35.5 |
| 91 | Terai-Duar Savannas and Grasslands | 2.9±0.9 | 2.7±1.0 | 3.1±0.9 | 7.5±21.2 | 71.0 | 11.1±24.7 | 71.0 | -6.7±14.3 | 37.1 |
| 92 | Llanos Savannas | 2.9±1.1 | 2.9±1.0 | 2.9±1.1 | -5.5±10.5 | 25.8 | 0.0±18.3 | 50.0 | -6.4±11.1 | 22.6 |
| 93 | Cerrado Woodlands and Savannas | 2.9±1.0 | 2.7±1.0 | 3.0±1.0 | 0.4±8.6 | 59.7 | 1.6±8.9 | 66.1 | -9.9±19.0 | 27.4 |
| 94 | Northern Prairies | 3.1±1.2 | 3.0±1.3 | 3.2±1.4 | 4.8±7.5 | 71.0 | 10.7±9.1 | 95.2 | 2.6±8.8 | 61.3 |
| 95 | Patagonian Steppe | 1.8±0.6 | 1.7±0.5 | 2.0±0.7 | -4.6±5.6 | 14.5 | -2.1±4.9 | 40.3 | -7.6±7.8 | 11.3 |
| 96 | Daurian/Mongolian Steppe | 3.3±1.2 | 3.1±1.2 | 3.5±1.4 | 9.9±5.7 | 96.8 | 7.5±6.3 | 90.3 | 19.9±8.5 | 100.0 |
| 97 | Sudd-Sahelian Flooded Grasslands  and Savannas | 2.8±0.9 | 2.9±1.0 | 2.8±0.9 | 1.0±8.1 | 59.7 | -0.4±8.3 | 53.2 | 6.7±13.6 | 62.9 |
| 98 | Zambezian Flooded Savannas | 2.9±1.0 | 2.8±1.0 | 3.0±1.0 | 1.1±5.4 | 59.7 | 2.1±5.3 | 61.3 | -10.8±14.8 | 24.2 |
| 99 | Rann of Kutch Flooded Grasslands | 2.8±0.8 | 2.4±0.7 | 3.2±1.0 | 21.1±59.0 | 67.7 | 22.5±69.2 | 66.1 | 20.3±45.6 | 58.1 |
| 100 | Everglades Flooded Grasslands | 2.1±0.8 | 2.3±0.8 | 2.0±0.7 | -4.0±10.8 | 41.9 | -10.7±15.7 | 22.6 | 3.3±13.1 | 58.1 |
| 101 | Pantanal Flooded Savannas | 3.0±1.1 | 2.8±1.1 | 3.2±1.1 | 0.2±9.2 | 66.1 | 1.4±10.2 | 66.1 | -6.4±22.6 | 35.5 |
| 102 | Ethiopian Highlands | 2.8±0.9 | 2.8±1.0 | 2.8±0.9 | 5.0±9.6 | 66.1 | 3.0±7.8 | 61.3 | 10.5±19.4 | 75.8 |
| 103 | Southern Rift Montane Woodlands | 2.7±0.9 | 2.6±0.9 | 2.8±1.0 | 5.3±8.7 | 77.4 | 6.4±9.2 | 77.4 | -9.0±26.0 | 29.0 |
| 104 | East African Moorlands | 2.5±0.8 | 2.6±0.9 | 2.5±0.8 | 9.4±10.6 | 87.1 | 9.9±11.6 | 88.7 | 9.2±11.5 | 85.5 |
| 105 | Drakensberg Montane Woodlands and  Grasslands | 2.6±0.8 | 2.5±0.8 | 2.7±0.8 | -1.3±6.7 | 37.1 | 0.2±7.0 | 48.4 | -5.3±9.3 | 25.8 |
| 106 | Central Range Subalpine Grasslands | 2.1±0.7 | 2.2±0.7 | 2.1±0.7 | 8.7±8.1 | 90.3 | 8.2±10.1 | 71.0 | 9.0±8.0 | 88.7 |
| 107 | Kinabalu Montane Shrublands | 2.1±0.7 | 2.1±0.7 | 2.0±0.7 | 4.6±7.0 | 75.8 | 7.0±9.5 | 77.4 | 2.4±7.9 | 64.5 |
| 108 | Northern Andean Paramo | 2.5±0.9 | 2.5±0.8 | 2.6±0.9 | 5.0±7.3 | 80.6 | 6.4±9.1 | 85.5 | 3.1±8.4 | 64.5 |
| 109 | Central Andean Dry Puna | 3.2±1.1 | 3.1±1.1 | 3.3±1.2 | -2.2±7.9 | 37.1 | -3.4±8.6 | 40.3 | 3.6±23.4 | 51.6 |
| 110 | Tibetan Plateau Steppe | 3.4±1.1 | 3.1±1.1 | 3.7±1.2 | 6.7±5.6 | 87.1 | 6.5±6.0 | 91.9 | 6.8±6.6 | 91.9 |
| 111 | Middle Asian Montane Woodlands  and Steppe | 3.3±1.1 | 3.1±1.1 | 3.5±1.2 | 1.2±5.5 | 56.5 | 8.1±7.5 | 93.5 | -8.6±10.7 | 22.6 |
| 112 | Eastern Himalayan Alpine Meadows | 3.3±1.1 | 2.8±1.0 | 3.7±1.3 | 8.8±16.1 | 77.4 | 12.5±19.6 | 79.0 | -1.4±12.5 | 50.0 |
| 113 | Alaskan North Slope Coastal Tundra | 4.8±1.7 | 3.8±1.4 | 5.7±2.1 | 21.6±10.1 | 100.0 | 20.1±9.8 | 100.0 | 24.7±14.0 | 98.4 |
| 114 | Canadian Low Arctic Tundra | 4.4±1.5 | 3.4±1.4 | 5.3±1.8 | 17.1±6.8 | 100.0 | 14.5±6.5 | 98.4 | 22.0±9.8 | 100.0 |
| 115 | Fenno-Scandia Alpine Tundra and  Taiga | 3.4±1.2 | 2.6±1.0 | 4.2±1.5 | 13.2±5.4 | 100.0 | 11.3±5.0 | 100.0 | 15.0±8.0 | 96.8 |
| 116 | Taimyr and Russian Coastal Tundra | 4.7±1.8 | 3.3±1.4 | 6.1±2.4 | 21.2±8.6 | 100.0 | 14.7±6.4 | 100.0 | 38.2±20.0 | 100.0 |
| 117 | Chukhote Coastal Tundra | 5.0±1.9 | 3.2±1.2 | 6.9±2.7 | 25.8±11.0 | 100.0 | 18.3±9.1 | 100.0 | 38.3±21.6 | 100.0 |
| 118 | Fynbos | 2.2±0.8 | 2.2±0.8 | 2.1±0.7 | -12.0±8.7 | 8.1 | -7.4±8.8 | 16.1 | -14.4±9.7 | 6.5 |
| 119 | Southwestern Australia Forests and  Scrub | 2.3±0.7 | 2.2±0.7 | 2.4±0.8 | -13.9±9.8 | 6.5 | -18.5±11.8 | 4.8 | -7.0±13.8 | 24.2 |
| 120 | Southern Australia Mallee and  Woodlands | 2.2±0.7 | 2.0±0.7 | 2.4±0.8 | -9.0±10.0 | 24.2 | -13.5±12.2 | 17.7 | -2.5±13.3 | 43.5 |
| 121 | California Chaparral and Woodlands | 2.5±0.9 | 2.4±0.8 | 2.7±1.0 | 1.1±15.6 | 45.2 | 2.5±16.0 | 46.8 | -9.9±17.7 | 25.8 |
| 122 | Chilean Matorral | 2.4±0.8 | 2.6±0.8 | 2.2±0.7 | -14.9±14.9 | 17.7 | -20.0±19.8 | 22.6 | -13.9±14.3 | 17.7 |
| 123 | Mediterranean Forests, Woodlands  and Scrub | 2.9±0.9 | 2.3±0.8 | 3.4±1.1 | -16.7±8.2 | 0.0 | -13.1±7.8 | 3.2 | -23.3±11.9 | 0.0 |
| 124 | Namib-Karoo-Kaokoveld Deserts and  Shrublands | 2.8±0.9 | 2.8±0.9 | 2.9±0.9 | -10.0±13.2 | 21.0 | -7.0±16.0 | 30.6 | -15.2±13.8 | 14.5 |
| 125 | Madagascar Spiny Thicket | 2.5±0.8 | 2.4±0.8 | 2.5±0.8 | -0.2±10.3 | 37.1 | 4.1±11.6 | 58.1 | -21.2±15.8 | 3.2 |
| 126 | Socotra Island Desert | 2.2±0.7 | 2.1±0.7 | 2.2±0.7 | 16.6±27.6 | 77.4 | 12.0±34.9 | 72.6 | 19.1±25.8 | 79.0 |
| 127 | Arabian Highlands Woodlands and  Shrublands | 2.9±0.9 | 2.9±1.0 | 2.8±0.9 | 6.3±24.6 | 51.6 | 6.7±26.9 | 48.4 | 8.3±30.3 | 56.5 |
| 128 | Carnavon Xeric Shrubs | 2.9±0.9 | 2.8±0.9 | 2.9±1.0 | -7.2±14.9 | 27.4 | -4.2±15.9 | 32.3 | -14.5±23.6 | 24.2 |
| 129 | Great Sandy-Tanami-Central Ranges  Desert | 3.0±1.0 | 3.0±1.0 | 3.0±1.0 | -5.5±13.2 | 30.6 | -2.6±13.5 | 41.9 | -14.6±23.9 | 17.7 |
| 130 | Sonoran-Baja Deserts | 2.7±0.9 | 2.7±0.9 | 2.7±0.9 | -5.0±14.1 | 30.6 | -3.6±14.5 | 35.5 | -7.2±22.5 | 32.3 |
| 131 | Chihuahuan-Tehuacán Deserts | 3.1±1.1 | 3.2±1.1 | 2.9±1.1 | -7.1±10.5 | 19.4 | -1.8±11.8 | 37.1 | -15.6±11.5 | 1.6 |
| 132 | Galapágos Islands Scrub | 2.2±0.7 | 2.2±0.7 | 2.2±0.8 | 25.1±24.4 | 88.7 | 29.3±29.3 | 87.1 | 20.5±25.1 | 77.4 |
| 133 | Atacama-Sechura Deserts | 2.8±1.0 | 2.7±0.9 | 2.9±1.0 | 7.0±8.5 | 77.4 | 7.2±8.6 | 82.3 | 6.7±16.2 | 67.7 |
| 134 | Central Asian Deserts | 3.1±1.0 | 2.8±1.1 | 3.3±1.1 | 2.0±8.4 | 64.5 | 5.8±8.3 | 72.6 | -9.1±14.9 | 33.9 |
| 135 | Gulf of Guinea Mangroves | 2.3±0.7 | 2.2±0.7 | 2.3±0.7 | 2.7±5.8 | 67.7 | 3.7±7.0 | 75.8 | 0.4±7.5 | 41.9 |
| 136 | East African Mangroves | 2.3±0.7 | 2.3±0.7 | 2.3±0.7 | 0.2±7.5 | 56.5 | 3.7±7.7 | 77.4 | -10.7±13.7 | 21.0 |
| 137 | Madagascar Mangroves | 2.2±0.7 | 2.2±0.7 | 2.2±0.7 | 1.5±8.2 | 58.1 | 3.4±9.0 | 67.7 | -17.8±17.9 | 8.1 |
| 138 | New Guinea Mangroves | 2.0±0.6 | 2.1±0.7 | 2.0±0.6 | 7.3±6.2 | 90.3 | 6.7±8.7 | 80.6 | 7.7±5.8 | 91.9 |
| 139 | Sundarbans Mangroves | 2.3±0.7 | 2.1±0.7 | 2.5±0.9 | 5.3±9.1 | 77.4 | 6.5±9.2 | 80.6 | -1.1±21.8 | 43.5 |
| 140 | Greater Sundas Mangroves | 2.1±0.7 | 2.1±0.7 | 2.1±0.7 | 4.1±5.7 | 82.3 | 5.0±6.4 | 85.5 | 3.3±6.2 | 74.2 |
| 141 | Amazon-Orinoco-Southern Caribbean  Mangroves | 2.3±0.7 | 2.3±0.7 | 2.3±0.8 | -6.1±13.3 | 25.8 | -2.1±15.5 | 45.2 | -9.5±13.4 | 16.1 |
| 142 | South American Pacific Mangroves | 2.2±0.7 | 2.2±0.7 | 2.2±0.7 | 4.7±8.2 | 77.4 | 5.5±9.0 | 74.2 | 4.3±9.5 | 72.6 |
| 147 | Amazon River and Flooded Forests | 2.9±1.1 | 2.7±1.1 | 3.1±1.3 | 1.6±9.4 | 62.9 | 3.5±9.5 | 69.4 | -2.4±14.2 | 50.0 |
| 143 | Congo River and Flooded Forests | 2.6±0.9 | 2.5±0.9 | 2.7±0.9 | 2.6±5.1 | 69.4 | 2.8±6.7 | 62.9 | 2.1±6.0 | 61.3 |
| 144 | Mekong River | 2.5±0.8 | 2.5±0.8 | 2.6±0.8 | 4.6±4.4 | 85.5 | 5.6±4.8 | 88.7 | 1.5±11.9 | 61.3 |
| 145 | Colorado River | 3.2±1.1 | 3.0±1.1 | 3.4±1.2 | -0.8±8.9 | 38.7 | 2.0±8.3 | 56.5 | -5.2±13.4 | 25.8 |
| 146 | Lower Mississippi River | 2.8±1.1 | 3.1±1.3 | 2.5±0.9 | -0.8±10.2 | 48.4 | -1.9±14.4 | 51.6 | 0.0±11.7 | 54.8 |
| 147 | Amazon River & Flooded Forests | 3.0±1.3 | 2.8±1.2 | 3.2±1.4 | 0.7±11.0 | 58.1 | 2.9±11.2 | 71.0 | -5.1±17.2 | 45.2 |
| 148 | Orinoco River & Flooded Forests | 2.9±1.1 | 2.9±1.1 | 2.9±1.1 | -3.3±9.9 | 37.1 | 2.6±18.8 | 51.6 | -4.7±10.2 | 35.5 |
| 149 | Yangtze River & Lakes | 2.8±0.9 | 2.7±0.9 | 2.9±1.0 | 6.6±5.2 | 87.1 | 7.0±5.1 | 93.5 | 5.6±9.0 | 72.6 |
| 150 | Congo Basin Piedmont Rivers &  Streams | 2.7±0.9 | 2.6±0.9 | 2.8±0.9 | 3.1±4.2 | 77.4 | 3.9±5.1 | 77.4 | 1.7±5.4 | 61.3 |
| 151 | Mississippi Piedmont Rivers &  Streams | 3.0±1.1 | 3.2±1.3 | 2.8±1.0 | 5.0±8.1 | 74.2 | 3.0±11.0 | 67.7 | 7.4±9.4 | 82.3 |
| 152 | Upper Amazon Rivers & Streams | 2.9±1.1 | 2.8±1.0 | 3.1±1.2 | 3.2±7.3 | 75.8 | 4.9±6.6 | 83.9 | -0.5±12.6 | 53.2 |
| 153 | Upper Paraná Rivers & Streams | 2.8±1.0 | 2.6±1.0 | 2.9±1.0 | 2.8±9.0 | 72.6 | 3.4±9.2 | 71.0 | 0.5±22.7 | 50.0 |
| 154 | Brazilian Shield Amazonian Rivers &  Streams | 3.0±1.1 | 2.7±1.1 | 3.3±1.2 | -0.2±8.8 | 54.8 | 1.4±8.8 | 61.3 | -9.4±20.4 | 33.9 |
| 155 | Niger River Delta | 2.3±0.7 | 2.2±0.7 | 2.3±0.8 | 3.0±6.5 | 66.1 | 4.4±7.5 | 72.6 | -1.1±9.4 | 45.2 |
| 156 | Indus River Delta | 2.8±0.8 | 2.4±0.8 | 3.1±0.9 | 16.8±47.3 | 66.1 | 23.5±82.1 | 66.1 | 30.5±73.0 | 61.3 |
| 157 | Volga River Delta | 3.0±1.1 | 3.1±1.1 | 2.9±1.1 | 0.7±7.3 | 56.5 | -10.0±14.1 | 25.8 | 6.5±7.6 | 82.3 |
| 158 | Mesopotamian Delta and Marshes | 3.3±1.0 | 2.9±0.9 | 3.7±1.1 | -8.7±19.3 | 22.6 | -13.5±16.2 | 14.5 | 0.1±46.1 | 43.5 |
| 159 | Danube River Delta | 3.0±1.0 | 2.7±1.0 | 3.3±1.2 | -7.8±8.9 | 14.5 | -2.4±9.1 | 40.3 | -13.3±12.4 | 16.1 |
| 160 | Lena River Delta | 4.7±1.8 | 3.1±1.3 | 6.3±2.5 | 23.1±9.5 | 100.0 | 16.6±7.8 | 96.8 | 41.5±23.0 | 100.0 |
| 161 | Upper Guinea Rivers & Streams | 2.5±0.8 | 2.4±0.8 | 2.6±0.9 | -0.1±8.4 | 48.4 | 0.1±8.4 | 53.2 | -2.7±12.9 | 33.9 |
| 162 | Madagascar Freshwater Ecosystem | 2.3±0.8 | 2.3±0.8 | 2.3±0.8 | -0.1±7.3 | 46.8 | 2.5±8.4 | 58.1 | -15.3±14.1 | 12.9 |
| 163 | Gulf of Guinea Rivers & Streams | 2.4±0.8 | 2.4±0.8 | 2.4±0.8 | 2.1±6.3 | 54.8 | 2.2±8.0 | 59.7 | 2.0±7.1 | 59.7 |
| 164 | Cape Rivers & Streams | 2.3±0.8 | 2.3±0.8 | 2.2±0.7 | -10.4±8.7 | 8.1 | -5.6±10.6 | 27.4 | -13.6±9.6 | 6.5 |
| 165 | New Guinea Rivers & Streams | 2.1±0.7 | 2.2±0.7 | 2.1±0.7 | 8.2±7.7 | 90.3 | 7.9±9.3 | 79.0 | 8.3±7.6 | 87.1 |
| 166 | New Caledonia Rivers & Streams | 1.9±0.6 | 1.9±0.6 | 1.8±0.6 | 0.6±10.7 | 43.5 | 2.8±12.6 | 53.2 | -4.7±12.7 | 33.9 |
| 167 | Kimberley Rivers & Streams | 2.8±0.9 | 2.6±0.9 | 2.9±0.9 | 0.1±8.4 | 56.5 | 1.0±8.4 | 61.3 | -6.4±25.3 | 43.5 |
| 168 | Southwest Australia Rivers & Streams | 2.2±0.7 | 2.1±0.7 | 2.3±0.8 | -14.5±9.6 | 4.8 | -18.2±11.3 | 3.2 | -7.7±13.0 | 21.0 |
| 169 | Eastern Australia Rivers & Streams | 2.5±0.8 | 2.4±0.8 | 2.6±0.9 | -4.2±9.0 | 35.5 | -11.5±10.8 | 16.1 | 0.3±10.1 | 48.4 |
| 170 | Xi Jiang Rivers & Streams | 2.5±0.8 | 2.4±0.8 | 2.5±0.9 | 5.7±6.8 | 77.4 | 7.7±6.9 | 85.5 | -2.1±14.1 | 46.8 |
| 171 | Western Ghats Rivers & Streams | 2.3±0.7 | 2.2±0.7 | 2.5±0.8 | 7.4±8.7 | 82.3 | 8.8±9.8 | 90.3 | 2.8±16.8 | 58.1 |
| 172 | Southwestern Sri Lanka Rivers | 2.0±0.7 | 2.0±0.7 | 2.0±0.7 | 10.2±9.0 | 91.9 | 14.6±12.5 | 93.5 | 3.9±11.0 | 64.5 |
| 173 | Salween River | 3.0±0.9 | 2.7±0.9 | 3.3±1.1 | 6.7±6.7 | 88.7 | 7.4±6.2 | 95.2 | 4.2±12.5 | 64.5 |
| 174 | Sundaland Rivers & Swamps | 2.2±0.7 | 2.2±0.7 | 2.1±0.7 | 5.3±6.1 | 80.6 | 5.9±6.2 | 85.5 | 4.9±7.1 | 75.8 |
| 175 | Southeastern Rivers & Streams | 2.6±0.9 | 2.8±1.1 | 2.3±0.8 | 1.9±8.2 | 66.1 | 0.4±11.3 | 61.3 | 3.3±9.7 | 67.7 |
| 176 | Pacific Northwest Coastal Rivers | 2.6±0.9 | 2.3±0.8 | 2.9±1.1 | 2.6±12.0 | 50.0 | 4.7±12.6 | 59.7 | -8.9±12.9 | 27.4 |
| 177 | Gulf of Alaska Coastal Rivers | 2.8±1.0 | 2.7±1.0 | 2.9±1.1 | 10.0±4.8 | 98.4 | 8.5±4.7 | 93.5 | 11.7±6.2 | 96.8 |
| 178 | Guianan Freshwater | 2.6±0.9 | 2.6±0.9 | 2.7±0.9 | -9.4±17.6 | 25.8 | -13.5±20.1 | 22.6 | -7.4±18.3 | 27.4 |
| 179 | Greater Antillean Freshwater | 2.1±0.7 | 2.1±0.7 | 2.0±0.7 | -10.4±12.6 | 17.7 | -17.8±15.7 | 9.7 | -1.6±10.5 | 45.2 |
| 180 | Balkan Rivers & Streams | 2.9±1.0 | 2.3±0.8 | 3.5±1.2 | -13.4±9.9 | 3.2 | -9.0±10.2 | 14.5 | -20.9±13.6 | 4.8 |
| 181 | Russian Far East Rivers & Wetlands | 3.5±1.3 | 3.0±1.2 | 4.0±1.5 | 11.5±4.8 | 100.0 | 9.7±4.3 | 100.0 | 15.9±8.0 | 98.4 |
| 182 | Rift Valley Lakes | 2.6±0.9 | 2.6±0.9 | 2.6±0.8 | 8.5±9.7 | 88.7 | 9.5±10.2 | 83.9 | 7.9±10.6 | 82.3 |
| 183 | High Andean Lakes | 3.1±1.1 | 3.1±1.0 | 3.1±1.1 | -1.7±7.8 | 35.5 | -3.0±8.1 | 38.7 | 2.7±17.6 | 54.8 |
| 184 | Lake Baikal | 3.5±1.4 | 3.1±1.3 | 3.8±1.6 | 11.5±5.3 | 96.8 | 7.1±6.5 | 90.3 | 23.6±10.4 | 100.0 |
| 185 | Lake Biwa | 2.5±0.9 | 2.4±0.9 | 2.5±0.9 | 5.4±7.0 | 83.9 | 6.9±7.1 | 87.1 | 2.8±9.5 | 58.1 |
| 186 | Cameroon Crater Lakes | 2.4±0.8 | 2.3±0.7 | 2.4±0.8 | 4.3±7.5 | 67.7 | 5.7±9.3 | 75.8 | 0.2±8.5 | 51.6 |
| 187 | Lakes Kutubu & Sentani | 2.2±0.7 | 2.2±0.7 | 2.1±0.7 | 8.7±8.2 | 87.1 | 8.2±9.9 | 77.4 | 8.8±8.7 | 83.9 |
| 188 | Central Sulawesi Lakes | 2.1±0.7 | 2.1±0.7 | 2.1±0.7 | 4.1±8.0 | 72.6 | 3.3±12.2 | 66.1 | 4.7±6.1 | 83.9 |
| 189 | Philippines Freshwater | 2.0±0.7 | 2.0±0.7 | 2.0±0.7 | 4.1±6.9 | 72.6 | 5.2±7.3 | 74.2 | 3.3±9.6 | 67.7 |
| 190 | Lake Inle | 2.6±0.8 | 2.4±0.8 | 2.9±0.9 | 5.9±9.3 | 71.0 | 7.3±8.9 | 77.4 | -4.4±26.0 | 43.5 |
| 191 | Yunnan Lakes & Streams | 2.5±0.8 | 2.5±0.8 | 2.6±0.8 | 6.1±9.1 | 67.7 | 7.1±9.0 | 79.0 | 3.1±14.8 | 53.2 |
| 192 | Mexican Highland Lakes | 2.7±1.0 | 2.8±1.0 | 2.6±1.0 | -8.9±11.1 | 14.5 | -6.0±12.0 | 35.5 | -15.9±12.9 | 4.8 |
| 193 | Central Australian Freshwater | 2.8±0.9 | 2.9±1.0 | 2.8±0.9 | -4.0±14.0 | 38.7 | 0.2±15.2 | 45.2 | -15.3±19.4 | 17.7 |
| 194 | Chihuahuan Freshwater | 3.1±1.1 | 3.2±1.1 | 2.9±1.1 | -7.0±9.9 | 19.4 | -2.1±11.3 | 38.7 | -14.1±11.2 | 4.8 |
| 195 | Anatolian Freshwater | 2.9±0.9 | 2.3±0.9 | 3.5±1.1 | -15.6±8.8 | 0.0 | -13.1±9.8 | 4.8 | -20.6±11.5 | 3.2 |
