# Supplementary material for: Global Priority Conservation Areas in the Face of 21st Century Climate Change
Source: PLoS One. 2013 Jan 24;8(1):e54839. doi: 10.1371/journal.pone.0054839 (PMC3554607; doi:10.1371/journal.pone.0054839)
Supplement: Table S2 — Observed and projected RCCI for 196 G200 ecoregions. The observed RCCI (RCCIobs) is based on differences in climate conditions between 1961−1980 and 1991−2009, generated from Climate Research Unit (CRU) TS 3.1 datasets; the projected RCCI is based on differences in climate conditions between 1991−2010 and 2081−2100, generated from an ensemble of 62 GCM × GHG emission scenario combinations. The relative magnitude of projected RCCI is measured by the multi-model mean RCCI (RCCImean) and the proportions of GCM × GHG emission scenario combinations with RCCI≥12 (the 50th percentile of RCCIobs) and RCCI≥16 (the 80th percentile of RCCIobs), respectively (abbreviated as Fr.(RCCI≥12) and Fr.(RCCI≥16)). (DOC) [file pone.0054839.s007.doc]

**Table S2 Observed and projected RCCI for 196 G200 ecoregions.** The observed RCCI (RCCI_obs_) is based on differences in climate observations between 1961−1980 and 1991−2009, generated from Climate Research Unit (CRU) TS 3.1 datasets; the projected RCCI is based on differences in climate conditions between 1991−2010 and 2081−2100, generated from an ensemble of 62 GCM × GHG emission scenario combinations. The relative magnitude of projected RCCI is measured by the multi-model mean RCCI (RCCI_mean_) and the proportions of GCM × GHG emission scenario combinations with RCCI≥12 (the 50^th^ percentile of RCCI_obs_) and RCCI≥16 (the 80^th^ percentile of RCCI_obs_), respectively (abbreviated as Fr.(RCCI≥12) and Fr.(RCCI≥16)).

| G200_ID | G200 Ecoregion | RCCI_obs_ | RCCI_mean_ | Fr. (RCCI≥12) (%) | Fr. (RCCI≥16) (%) |
| --- | --- | --- | --- | --- | --- |
| 1 | Guinean Moist Forests | 11 | 13.9 | 72.6 | 35.5 |
| 2 | Congolian Coastal Forests | 5 | 13.4 | 69.4 | 30.6 |
| 3 | Cameroon Highlands Forests | 12 | 13.9 | 83.9 | 37.1 |
| 4 | Northeastern Congo Basin Moist  Forests | 16 | 13.6 | 77.4 | 35.5 |
| 5 | Central Congo Basin Moist Forests | 11 | 13.5 | 75.8 | 30.6 |
| 6 | Western Congo Basin Moist Forests | 10 | 12.9 | 67.7 | 27.4 |
| 7 | Albertine Rift Montane Forests | 13 | 13.7 | 67.7 | 38.7 |
| 8 | East African Coastal Forests | 16 | 15.1 | 74.2 | 43.5 |
| 9 | Eastern Arc Montane Forests | 9 | 14.5 | 79.0 | 41.9 |
| 10 | Madagascar Forests and Shrublands | 18 | 14.3 | 69.4 | 37.1 |
| 11 | Seychelles and Mascarenes Moist  Forests | 14 | 13.7 | 72.6 | 33.9 |
| 12 | Sulawesi Moist Forests | 14 | 14.2 | 74.2 | 32.3 |
| 13 | Moluccas Moist Forests | 8 | 14.0 | 75.8 | 38.7 |
| 14 | Southern New Guinea Lowland  Forests | 16 | 13.7 | 71.0 | 32.3 |
| 15 | New Guinea Montane Forests | 14 | 14.2 | 75.8 | 40.3 |
| 16 | Solomons-Vanuatu-Bismarck Moist  Forests | 16 | 13.6 | 64.5 | 32.3 |
| 17 | Queensland Tropical Forests | 19 | 14.4 | 69.4 | 40.3 |
| 18 | New Caledonia Moist Forests | 10 | 14.0 | 69.4 | 33.9 |
| 19 | Lord Howe and Norfolk Island  Forests | 12 | 14.9 | 74.2 | 50.0 |
| 20 | Southwestern Ghats Moist Forest | 14 | 14.8 | 88.7 | 40.3 |
| 21 | Sri Lankan Moist Forest | 6 | 15.1 | 80.6 | 50.0 |
| 22 | North Indochina Subtropical Moist  Forests | 16 | 14.6 | 79.0 | 50.0 |
| 23 | Southeast China-Hainan Moist  Forests | 7 | 13.4 | 64.5 | 29.0 |
| 24 | Taiwan Montane Forests | 10 | 13.6 | 72.6 | 25.8 |
| 25 | Annamite Range Moist Forests | 13 | 14.0 | 74.2 | 37.1 |
| 26 | Sumatran Islands Lowland and  Montane Forests | 12 | 13.0 | 74.2 | 29.0 |
| 27 | Philippines Moist Forests | 8 | 13.3 | 72.6 | 27.4 |
| 28 | Palawan Moist Forests | 11 | 14.4 | 80.6 | 38.7 |
| 29 | Kayah-Karen/Tenasserim Moist  Forests | 16 | 13.3 | 71.0 | 25.8 |
| 30 | Peninsular Malaysia Lowland and  Montane Forests | 10 | 12.9 | 67.7 | 27.4 |
| 31 | Borneo Lowland and Montane Forests | 11 | 14.1 | 74.2 | 33.9 |
| 32 | Nansei Shoto Archipelago Forests | 10 | 12.9 | 64.5 | 35.5 |
| 33 | Eastern Deccan Plateau Moist Forests | 14 | 16.3 | 85.5 | 51.6 |
| 34 | Naga-Manapuri-Chin Hills Moist  Forests | 8 | 14.5 | 77.4 | 45.2 |
| 35 | Cardamom Mountains Moist Forests | 10 | 13.1 | 59.7 | 32.3 |
| 36 | Western Java Montane Forests | 10 | 13.6 | 69.4 | 35.5 |
| 37 | Greater Antillean Moist Forests | 15 | 15.6 | 83.9 | 53.2 |
| 38 | Talamancan-Isthmian Pacific Forests | 18 | 16.7 | 82.3 | 56.5 |
| 39 | Chocó-Darién Moist Forests | 11 | 13.5 | 69.4 | 32.3 |
| 40 | Northern Andean Montane Forests | 10 | 13.3 | 67.7 | 24.2 |
| 41 | Coastal Venezuela Montane Forests | 12 | 17.4 | 88.7 | 67.7 |
| 42 | Guianan Moist Forests | 8 | 15.8 | 85.5 | 50.0 |
| 43 | Napo Moist Forests | 5 | 14.5 | 71.0 | 46.8 |
| 44 | Rio Negro-Juruá Moist Forests | 13 | 15.3 | 83.9 | 46.8 |
| 45 | Guianan Highlands Moist Forests | 12 | 15.9 | 75.8 | 50.0 |
| 46 | Central Andean Yungas | 6 | 13.5 | 75.8 | 30.6 |
| 47 | Southwestern Amazonian Moist  Forests | 14 | 15.1 | 77.4 | 50.0 |
| 48 | Atlantic Forests | 12 | 13.7 | 75.8 | 37.1 |
| 49 | Southern Pacific Islands Forests | 8 | 14.2 | 75.8 | 38.7 |
| 50 | Hawaii Moist Forest | 12 | 15.3 | 85.5 | 59.7 |
| 51 | Madagascar Dry Forests | 16 | 13.8 | 67.7 | 37.1 |
| 52 | Nusu Tenggara Dry Forests | 12 | 14.3 | 82.3 | 33.9 |
| 53 | New Caledonia Dry Forests | 7 | 14.2 | 72.6 | 38.7 |
| 54 | Indochina Dry Forests | 13 | 13.4 | 71.0 | 27.4 |
| 55 | Chhota-Nagpur Dry Forests | 19 | 15.8 | 83.9 | 50.0 |
| 56 | Southern Mexican Dry Forests | 12 | 15.9 | 83.9 | 53.2 |
| 57 | Tumbesian-Andean Valleys Dry  Forests | 9 | 14.2 | 77.4 | 41.9 |
| 58 | Chiquitano Dry Forests | 11 | 15.5 | 77.4 | 46.8 |
| 59 | Atlantic Dry Forests | 14 | 16.4 | 90.3 | 56.5 |
| 60 | Hawaii Dry Forests | 10 | 15.4 | 83.9 | 61.3 |
| 61 | Sierra Madre Oriental and Occidental  Pine-Oak Forests | 10 | 16.3 | 87.1 | 51.6 |
| 62 | Greater Antillean Pine Forests | 14 | 14.6 | 80.6 | 40.3 |
| 63 | Mesoamerican Pine-Oak Forests | 9 | 15.2 | 75.8 | 46.8 |
| 64 | Eastern Asutralia Temperate Forests | 18 | 14.6 | 77.4 | 41.9 |
| 65 | Tasmanian Temperate Rainforests | 10 | 13.3 | 66.1 | 33.9 |
| 66 | New Zealand Temperate Forests | 5 | 11.3 | 46.8 | 12.9 |
| 67 | Eastern Himalayan Broadleaf and  Conifer Forests | 7 | 15.9 | 82.3 | 62.9 |
| 68 | Western Himalayan Temperate  Forests | 10 | 16.0 | 79.0 | 59.7 |
| 69 | Appalachian and Mixed Mesophytic  Forests | 13 | 13.7 | 74.2 | 25.8 |
| 70 | Southwest China Temperate Forests | 7 | 13.3 | 67.7 | 35.5 |
| 71 | Russian Far East Broadleaf and  Mixed Forests | 13 | 14.1 | 72.6 | 38.7 |
| 72 | Pacific Temperate Rainforests | 10 | 13.4 | 71.0 | 37.1 |
| 73 | Klamath-Siskiyou Coniferous Forests | 9 | 13.8 | 72.6 | 32.3 |
| 74 | Sierra Nevada Coniferous Forests | 12 | 14.7 | 79.0 | 43.5 |
| 75 | Southeastern Conifer and Broadleaf  Forests | 13 | 13.7 | 69.4 | 33.9 |
| 76 | Valdivian Temperate Rain Forests  / Juan Fernández | 13 | 14.4 | 83.9 | 41.9 |
| 77 | European-Mediterranean Montane  Forests | 13 | 15.0 | 79.0 | 45.2 |
| 78 | Caucasus-Anatolian-Hyrcanian Temperate Forests | 10 | 15.8 | 87.1 | 48.4 |
| 79 | Altai-Sayan Montane Forests | 8 | 15.7 | 85.5 | 54.8 |
| 80 | Hengduan Shan Conifer Forests | 10 | 14.0 | 69.4 | 30.6 |
| 81 | Muskwa/Slave Lake Boreal Forests | 15 | 16.0 | 83.9 | 54.8 |
| 82 | Canadian Boreal Taiga | 13 | 17.7 | 95.2 | 67.7 |
| 83 | Ural Mountains Taiga and Tundra | 17 | 18.1 | 95.2 | 79.0 |
| 84 | Central and Eastern Siberian Taiga | 18 | 18.6 | 91.9 | 83.9 |
| 85 | Kamchatka Taiga and Grasslands | 14 | 17.5 | 91.9 | 64.5 |
| 86 | Horn of Africa Acacia Savannas | 16 | 16.4 | 91.9 | 53.2 |
| 87 | East African Acacia Savannas | 14 | 15.4 | 75.8 | 48.4 |
| 88 | Central and Eastern Miombo  Woodlands | 8 | 14.5 | 85.5 | 33.9 |
| 89 | Sudanian Savannas | 5 | 14.5 | 79.0 | 45.2 |
| 90 | Northern Australia and Trans-Fly  Savannas | 5 | 14.3 | 66.1 | 45.2 |
| 91 | Terai-Duar Savannas and Grasslands | 10 | 16.8 | 87.1 | 64.5 |
| 92 | Llanos Savannas | 14 | 15.3 | 83.9 | 43.5 |
| 93 | Cerrado Woodlands and Savannas | 12 | 14.9 | 75.8 | 38.7 |
| 94 | Northern Prairies | 14 | 14.6 | 72.6 | 48.4 |
| 95 | Patagonian Steppe | 11 | 12.9 | 66.1 | 30.6 |
| 96 | Daurian/Mongolian Steppe | 17 | 16.1 | 83.9 | 56.5 |
| 97 | Sudd-Sahelian Flooded Grasslands  and Savannas | 26 | 14.5 | 77.4 | 43.5 |
| 98 | Zambezian Flooded Savannas | 16 | 14.3 | 72.6 | 43.5 |
| 99 | Rann of Kutch Flooded Grasslands | 10 | 17.0 | 91.9 | 64.5 |
| 100 | Everglades Flooded Grasslands | 20 | 14.3 | 77.4 | 46.8 |
| 101 | Pantanal Flooded Savannas | 9 | 15.5 | 75.8 | 46.8 |
| 102 | Ethiopian Highlands | 12 | 14.4 | 79.0 | 40.3 |
| 103 | Southern Rift Montane Woodlands | 12 | 15.4 | 82.3 | 43.5 |
| 104 | East African Moorlands | 12 | 14.6 | 77.4 | 46.8 |
| 105 | Drakensberg Montane Woodlands and  Grasslands | 18 | 13.1 | 64.5 | 30.6 |
| 106 | Central Range Subalpine Grasslands | 12 | 14.6 | 74.2 | 46.8 |
| 107 | Kinabalu Montane Shrublands | 14 | 14.3 | 75.8 | 41.9 |
| 108 | Northern Andean Paramo | 7 | 13.8 | 77.4 | 33.9 |
| 109 | Central Andean Dry Puna | 2 | 14.5 | 72.6 | 40.3 |
| 110 | Tibetan Plateau Steppe | 8 | 13.7 | 69.4 | 38.7 |
| 111 | Middle Asian Montane Woodlands  and Steppe | 16 | 14.5 | 69.4 | 48.4 |
| 112 | Eastern Himalayan Alpine Meadows | 3 | 16.0 | 87.1 | 53.2 |
| 113 | Alaskan North Slope Coastal Tundra | 18 | 21.3 | 100.0 | 88.7 |
| 114 | Canadian Low Arctic Tundra | 23 | 19.8 | 98.4 | 85.5 |
| 115 | Fenno-Scandia Alpine Tundra and  Taiga | 20 | 17.5 | 90.3 | 67.7 |
| 116 | Taimyr and Russian Coastal Tundra | 18 | 21.3 | 100.0 | 96.8 |
| 117 | Chukhote Coastal Tundra | 5 | 21.6 | 100.0 | 96.8 |
| 118 | Fynbos | 16 | 14.9 | 74.2 | 53.2 |
| 119 | Southwestern Australia Forests and  Scrub | 14 | 15.8 | 85.5 | 59.7 |
| 120 | Southern Australia Mallee and  Woodlands | 14 | 15.1 | 82.3 | 53.2 |
| 121 | California Chaparral and Woodlands | 15 | 15.1 | 85.5 | 45.2 |
| 122 | Chilean Matorral | 12 | 16.5 | 87.1 | 56.5 |
| 123 | Mediterranean Forests, Woodlands  and Scrub | 14 | 17.9 | 93.5 | 77.4 |
| 124 | Namib-Karoo-Kaokoveld Deserts and  Shrublands | 12 | 15.9 | 77.4 | 56.5 |
| 125 | Madagascar Spiny Thicket | 14 | 15.0 | 80.6 | 53.2 |
| 126 | Socotra Island Desert | 14 | 16.7 | 88.7 | 62.9 |
| 127 | Arabian Highlands Woodlands and  Shrublands | 15 | 15.6 | 83.9 | 58.1 |
| 128 | Carnavon Xeric Shrubs | 14 | 16.5 | 85.5 | 61.3 |
| 129 | Great Sandy-Tanami-Central Ranges  Desert | 9 | 15.7 | 80.6 | 54.8 |
| 130 | Sonoran-Baja Deserts | 12 | 15.7 | 80.6 | 51.6 |
| 131 | Chihuahuan-Tehuacán Deserts | 6 | 15.8 | 90.3 | 54.8 |
| 132 | Galapágos Islands Scrub | 24 | 16.9 | 85.5 | 66.1 |
| 133 | Atacama-Sechura Deserts | 12 | 15.1 | 80.6 | 46.8 |
| 134 | Central Asian Deserts | 18 | 15.1 | 82.3 | 50.0 |
| 135 | Gulf of Guinea Mangroves | 13 | 13.1 | 71.0 | 25.8 |
| 136 | East African Mangroves | 16 | 14.0 | 71.0 | 35.5 |
| 137 | Madagascar Mangroves | 19 | 14.3 | 75.8 | 41.9 |
| 138 | New Guinea Mangroves | 11 | 13.8 | 66.1 | 32.3 |
| 139 | Sundarbans Mangroves | 16 | 14.8 | 77.4 | 41.9 |
| 140 | Greater Sundas Mangroves | 11 | 13.9 | 74.2 | 40.3 |
| 141 | Amazon-Orinoco-Southern Caribbean  Mangroves | 10 | 15.2 | 85.5 | 51.6 |
| 142 | South American Pacific Mangroves | 9 | 13.4 | 69.4 | 37.1 |
| 147 | Amazon River and Flooded Forests | 11 | 13.1 | 66.1 | 29.0 |
| 143 | Congo River and Flooded Forests | 11 | 13.1 | 69.4 | 32.3 |
| 144 | Mekong River | 10 | 14.0 | 77.4 | 32.3 |
| 145 | Colorado River | 18 | 14.1 | 75.8 | 33.9 |
| 146 | Lower Mississippi River | 14 | 15.4 | 82.3 | 45.2 |
| 147 | Amazon River & Flooded Forests | 11 | 16.5 | 87.1 | 54.8 |
| 148 | Orinoco River & Flooded Forests | 10 | 14.9 | 79.0 | 45.2 |
| 149 | Yangtze River & Lakes | 6 | 12.4 | 61.3 | 19.4 |
| 150 | Congo Basin Piedmont Rivers &  Streams | 14 | 13.1 | 67.7 | 32.3 |
| 151 | Mississippi Piedmont Rivers &  Streams | 16 | 13.8 | 74.2 | 33.9 |
| 152 | Upper Amazon Rivers & Streams | 7 | 14.3 | 80.6 | 37.1 |
| 153 | Upper Paraná Rivers & Streams | 15 | 14.3 | 74.2 | 43.5 |
| 154 | Brazilian Shield Amazonian Rivers &  Streams | 12 | 14.9 | 75.8 | 45.2 |
| 155 | Niger River Delta | 13 | 13.1 | 67.7 | 33.9 |
| 156 | Indus River Delta | 12 | 17.2 | 93.5 | 66.1 |
| 157 | Volga River Delta | 18 | 14.4 | 66.1 | 40.3 |
| 158 | Mesopotamian Delta and Marshes | 16 | 17.4 | 91.9 | 64.5 |
| 159 | Danube River Delta | 13 | 15.7 | 85.5 | 48.4 |
| 160 | Lena River Delta | 15 | 20.7 | 100.0 | 90.3 |
| 161 | Upper Guinea Rivers & Streams | 8 | 13.9 | 72.6 | 35.5 |
| 162 | Madagascar Freshwater Ecosystem | 18 | 14.2 | 74.2 | 35.5 |
| 163 | Gulf of Guinea Rivers & Streams | 3 | 13.3 | 67.7 | 30.6 |
| 164 | Cape Rivers & Streams | 16 | 14.8 | 75.8 | 48.4 |
| 165 | New Guinea Rivers & Streams | 14 | 14.6 | 72.6 | 45.2 |
| 166 | New Caledonia Rivers & Streams | 9 | 14.1 | 71.0 | 37.1 |
| 167 | Kimberley Rivers & Streams | 11 | 14.0 | 66.1 | 41.9 |
| 168 | Southwest Australia Rivers & Streams | 16 | 15.8 | 80.6 | 56.5 |
| 169 | Eastern Australia Rivers & Streams | 15 | 14.8 | 87.1 | 38.7 |
| 170 | Xi Jiang Rivers & Streams | 8 | 13.7 | 72.6 | 29.0 |
| 171 | Western Ghats Rivers & Streams | 11 | 14.5 | 77.4 | 38.7 |
| 172 | Southwestern Sri Lanka Rivers | 5 | 15.1 | 80.6 | 50.0 |
| 173 | Salween River | 10 | 14.1 | 75.8 | 33.9 |
| 174 | Sundaland Rivers & Swamps | 9 | 14.0 | 72.6 | 38.7 |
| 175 | Southeastern Rivers & Streams | 12 | 14.2 | 75.8 | 41.9 |
| 176 | Pacific Northwest Coastal Rivers | 7 | 14.0 | 75.8 | 32.3 |
| 177 | Gulf of Alaska Coastal Rivers | 14 | 13.9 | 72.6 | 33.9 |
| 178 | Guianan Freshwater | 6 | 15.8 | 82.3 | 50.0 |
| 179 | Greater Antillean Freshwater | 16 | 15.1 | 80.6 | 50.0 |
| 180 | Balkan Rivers & Streams | 10 | 16.9 | 80.6 | 59.7 |
| 181 | Russian Far East Rivers & Wetlands | 12 | 17.0 | 98.4 | 59.7 |
| 182 | Rift Valley Lakes | 9 | 15.1 | 82.3 | 46.8 |
| 183 | High Andean Lakes | 6 | 14.6 | 72.6 | 48.4 |
| 184 | Lake Baikal | 18 | 16.0 | 82.3 | 53.2 |
| 185 | Lake Biwa | 21 | 12.6 | 62.9 | 21.0 |
| 186 | Cameroon Crater Lakes | 13 | 13.6 | 72.6 | 30.6 |
| 187 | Lakes Kutubu & Sentani | 13 | 14.3 | 71.0 | 35.5 |
| 188 | Central Sulawesi Lakes | 12 | 14.0 | 72.6 | 33.9 |
| 189 | Philippines Freshwater | 8 | 13.7 | 66.1 | 45.2 |
| 190 | Lake Inle | 20 | 14.6 | 74.2 | 50.0 |
| 191 | Yunnan Lakes & Streams | 12 | 15.1 | 77.4 | 50.0 |
| 192 | Mexican Highland Lakes | 8 | 15.5 | 82.3 | 46.8 |
| 193 | Central Australian Freshwater | 18 | 15.6 | 87.1 | 46.8 |
| 194 | Chihuahuan Freshwater | 8 | 15.5 | 87.1 | 56.5 |
| 195 | Anatolian Freshwater | 14 | 17.7 | 88.7 | 71.0 |
