# Supplementary material for: Global Priority Conservation Areas in the Face of 21st Century Climate Change
Source: PLoS One. 2013 Jan 24;8(1):e54839. doi: 10.1371/journal.pone.0054839 (PMC3554607; doi:10.1371/journal.pone.0054839)
Supplement: Table S3 — Value of the factor n in the definition of the RCCI. Any change in each climatic factor is assigned an integer value “n” between 0 and 4 according to the absolute value of change. Note that small changes below a certain threshold do not contribute to the index (n = 0) and that larger changes are weighted more heavily (i.e., the factor n doubles from each category to the next). This table is adapted from Table 1 of Ref. [6]. (DOC) [file pone.0054839.s008.doc]

**Table S3 Value of the factor n in the definition of the RCCI.** Any change in each climatic factor is assigned an integer value “n” between 0 and 4 according to the absolute value of change. Note that small changes below a certain threshold do not contribute to the index (n = 0) and that larger changes are weighted more heavily (i.e., the factor n doubles from each category to the next). This table is adapted from Table 1 of Ref. [S1].

| n | |ΔP| | |Δσ_P_| | |RWAF| | |Δσ_T_| |
| --- | --- | --- | --- | --- |
| 0 | < 5% | < 5% | < 1.1 | < 5% |
| 1 | 5 − 10% | 5 − 10% | 1.1 − 1.3 | 5 − 10% |
| 2 | 10 − 15% | 10 − 20% | 1.3 − 1.5 | 10 − 15% |
| 4 | > 15% | > 20% | > 1.5 | > 15% |
