# Supplementary material for: Global Priority Conservation Areas in the Face of 21st Century Climate Change
Source: PLoS One. 2013 Jan 24;8(1):e54839. doi: 10.1371/journal.pone.0054839 (PMC3554607; doi:10.1371/journal.pone.0054839)
Supplement: Table S4 — The ensemble of 23 General Circulation Models (GCMs) used in this study. Figures in brackets indicate the number of different realizations for a GCM × GHG emission scenario combination. (DOC) [file pone.0054839.s009.doc]

**Table S4 The ensemble of 23 General Circulation Models (GCMs) used in this study.** Figures in brackets indicate the number of different realizations for a GCM × GHG emission scenario combination.

| Model | 20c3m | A1B | A2 | B1 |
| --- | --- | --- | --- | --- |
| bccr_bcm2_0 | 1(1) | 1(1) | 1(1) | 1(1) |
| cccma_cgcm3_1 | 1(5) | 1(5) | 1(5) | 1(5) |
| cccma_cgcm3_1_t63 | 1(1) | 1(1) | 0(0) | 1(1) |
| cnrm_cm3 | 1(1) | 1(1) | 1(1) | 1(1) |
| csiro_mk3_0 | 1(3) | 1(1) | 1(1) | 1(1) |
| csiro_mk3_5 | 1(3) | 1(1) | 1(1) | 1(1) |
| gfdl_cm2_0 | 1(3) | 1(1) | 1(1) | 1(1) |
| gfdl_cm2_1 | 1(3) | 1(1) | 1(1) | 1(1) |
| giss_aom | 1(2) | 1(2) | 0(0) | 1(2) |
| giss_model_e_h | 1(5) | 1(3) | 0(0) | 0(0) |
| giss_model_e_r | 1(9) | 1(5) | 1(1) | 1(1) |
| iap_fgoals1_0_g | 1(3) | 1(3) | 0(0) | 1(3) |
| ingv_echam4 | 1(1) | 1(1) | 1(1) | 0(0) |
| inmcm3_0 | 1(1) | 1(1) | 1(1) | 1(1) |
| ipsl_cm4 | 1(2) | 1(1) | 1(1) | 1(1) |
| miroc3_2_medres | 1(3) | 1(3) | 1(3) | 1(3) |
| miub_echo_g | 1(5) | 1(3) | 1(3) | 1(3) |
| mpi_echam5 | 1(4) | 1(4) | 1(3) | 1(3) |
| mri_cgcm2_3_2a | 1(5) | 1(5) | 1(5) | 1(5) |
| ncar_ccsm3_0 | 1(8) | 1(7) | 1(4) | 1(9) |
| ncar_pcm1 | 1(4) | 1(4) | 1(4) | 1(2) |
| ukmo_hadcm3 | 1(2) | 1(1) | 1(1) | 1(1) |
| ukmo_hadgem1 | 1(2) | 1(1) | 1(1) | 0(0) |
